# Surrogate *in vitro* activation of innate immunity synergizes with interleukin-7 to unleash rapid antigen-driven outgrowth of CD4+ and CD8+ human peripheral blood T-cells naturally recognizing MUC1, HER2/neu and other tumor-associated antigens

**DOI:** 10.18632/oncotarget.13911

**Published:** 2016-12-11

**Authors:** Latha B. Pathangey, Dustin B. McCurry, Sandra J. Gendler, Ana L. Dominguez, Jessica E. Gorman, Girish Pathangey, Laurie A. Mihalik, Yushe Dang, Mary L. Disis, Peter A. Cohen

**Affiliations:** ^1^ Department of Biochemistry and Molecular Biology, Mayo Clinic, Scottsdale, AZ, USA; ^2^ Department of Immunology, Mayo Clinic, Scottsdale, AZ, USA; ^3^ Department of Hematology and Oncology, Mayo Clinic, Scottsdale, AZ, USA; ^4^ Tumor Vaccine Group, Center for Translational Medicine in Womens Health, University of Washington, Seattle, WA, USA

**Keywords:** PBMC, TLR agonists, GM-CSF, IL-7, adoptive therapy, MUC1

## Abstract

Effective adoptive immunotherapy has proved elusive for many types of human cancer, often due to difficulties achieving robust expansion of natural tumor-specific T-cells from peripheral blood. We hypothesized that antigen-driven T-cell expansion might best be triggered *in vitro* by acute activation of innate immunity to mimic a life-threatening infection. Unfractionated peripheral blood mononuclear cells (PBMC) were subjected to a two-step culture, first synchronizing their exposure to exogenous antigens with aggressive surrogate activation of innate immunity, followed by ?-chain cytokine-modulated T-cell hyperexpansion. Step 1 exposure to GM-CSF plus paired Toll-like receptor agonists (resiquimod and LPS), stimulated abundant IL-12 and IL-23 secretion, as well as upregulated co-stimulatory molecules and CD11c expression within the myeloid (CD33+) subpopulation. Added synthetic long peptides (>20aa) derived from widely expressed oncoproteins (MUC1, HER2/neu and CMVpp65), were reliably presented to CD4+ T-cells and cross-presented to CD8+ T-cells. Both presentation and cross-presentation demonstrated proteasomal and Sec61 dependence that could bypass the endoplasmic reticulum. Step 2 exposure to exogenous IL-7 or IL-7+IL-2 produced selective and sustained expansion of both CD4+ and CD8+ peptide-specific T-cells with a predominant interferon-?-producing T1-type, as well as the antigen-specific ability to lyse tumor targets. Other ?-chain cytokines and/or combinations were initially proliferogenic, but followed by a contractile phase not observed with IL-7 or IL-7+IL-2. Regulatory T-cells were minimally propagated under these culture conditions. This mechanistically rational culture sequence, effective even for unvaccinated donors, enables rapid preparation of T-cells recognizing tumor-associated antigens expressed by the majority of human cancers, including pancreatic cancers, breast cancers and glioblastomas.

## INTRODUCTION

Adoptive immunotherapy (AIT) employing autologous culture-expanded T-cells is a promising strategy for cancer patients. Patients with advanced melanoma often experience durable tumor regressions when they are reinfused with culture-expanded natural T-cells derived from their own tumor nodules, in conjunction with nonmyeloablative chemotherapy and high dose IL-2 [1, 2]. Such AIT with naturally occurring tumor-infiltrating lymphocytes (TIL) has recently been extended to target individual mutations in a variety of cancers besides melanoma [3, 4]. Responsiveness to AIT correlates to the ability of culture-expanded TIL to produce interferon- γ (IFNγ) upon tumor reexposure, as well as to the dual presence of CD4+ and CD8+ T-cells in the administered AIT product [5–7]. Consistent with these results, we and others have shown that tumor-specific CD4+ T-cells are sometimes a required component of curative AIT in syngeneic mouse tumor models, and that co-transfer of IFNγ-producing anti-tumor CD4+ and CD8+ T cells can markedly accelerate tumor rejection [8–11].

Despite these promising developments, significant barriers have impeded the wider use of natural T-lymphocytes for AIT. It has been observed that TIL, including mutation-specific TIL, can only be culture-expanded from a subset of resected tumor nodules, and equivalently performing T-cells have yet to be reliably expanded to large numbers from human peripheral blood mononuclear cells (PBMC) [12, 13]. The recent widespread testing of lymphocytes transfected to express alternative T-cell receptors or chimeric antigen receptors (CAR) reflects, paradoxically, that it currently remains easier to gene-modify peripheral lymphocytes than it is to propagate natural tumor-reactive T-lymphocytes from PBMC [14–18].

Peripheral blood nonetheless constitutes the most convenient compartment, and often the only readily available compartment, from which to harvest human T-cells for culture expansion. Billions of PBMC can reliably be collected by leukapheresis regardless of a patient's tumor type, containing not only CD4^+^ and CD8^+^ T-cells, but also monocytes which can be differentiated into professional antigen (Ag)-presenting dendritic cells (DCs) [19–21].

Despite the historic difficulties propagating natural tumor-reactive T-cells from PBMC, it has recently proved possible to culture-expand PBMC-derived T-cells from lymphoma patients which recognize Epstein-Barr virus (EBV) tumor-associated epitopes. Such cultured EBV-specific T-cells are often therapeutically active when reinfused into patients [22]. Similarly, we have shown that unfractionated PBMC from HER2/neu-vaccinated cancer patients can give rise to enriched HER2-specific CD4+ and CD8+ T-cells, when such PBMC are cultured with the vaccination peptides as well as with exogenous recombinant IL-12 and IL-2, then further expanded by polyclonal anti-CD3/CD28 stimulation [23, 24]. When such T-cells are reinfused into patients after administration of cyclophosphamide, they often persist for months in the peripheral circulation, and exert dose-dependent anti-tumor effects which are proportionate to the number of HER2-specific T-cells reinfused [24]. Notably, the inclusion of exogenous IL-12 in culture was required to engage the selective outgrowth of HER2-specific T-cells [23].

We hypothesized that upfront exposure of unfractionated PBMCs to aggressive activators of innate immunity, rather than to exogenous IL-12, could optimally trigger the differentiation of IL-12-secreting, type-1 polarized myeloid DCs naturally contained within the myeloid PBMC subpopulation. We hypothesized that such *in vitro* simulation of a life threatening infection could further enhance the processing and presentation of exogenous tumor-associated Ags added to culture, promoting a highly desirable expansion of both CD4^+^ and CD8^+^ T1-type (IFNγ-secreting), Ag-specific T-cells also present in unfractionated PBMC.

Myeloid DC as well as monocytic DC precursors are maximally activated by synergizing combinations of closely timed danger signals [25–27]. Synergistic danger signal pairings vary by species, and can consist of a MyD88-activating and a TRIF-activating Toll-like receptor (TLR) agonist, a single TLR agonist plus IFNγ or CD40 ligand, or the combination of IFNγ plus CD40 ligand itself [25, 28]. Successful triggering of this alarm system results in activation of innate immunity, type 1 polarization of myeloid DC, and emblematic production of IL-12 and IL-23 to enhance T1- and T17-type responses against life-threatening pathogens [25, 29]. This tightly regulated requirement for multiple closely timed danger signals protects the host from mounting potentially self-destructive immune responses against isolated false alarms. We postulated, however, that such maximally synergistic danger signals might safely and conveniently be applied to unfractionated PBMC outside the body in order to strongly activate innate immunity, license acquired immunity, and ramp up *in vitro* sensitization of natural T-cells, both CD4+ and CD8+, to tumor-associated antigens.

## RESULTS

### Combined upfront conditioning of unfractionated human PBMC with recombinant GM-CSF, resiquimod, and LPS licenses robust IL-12 production, costimulatory molecule upregulation within the myeloid subset, and markedly enhanced expansion of Ag-driven T-cells

**Figure 1:**
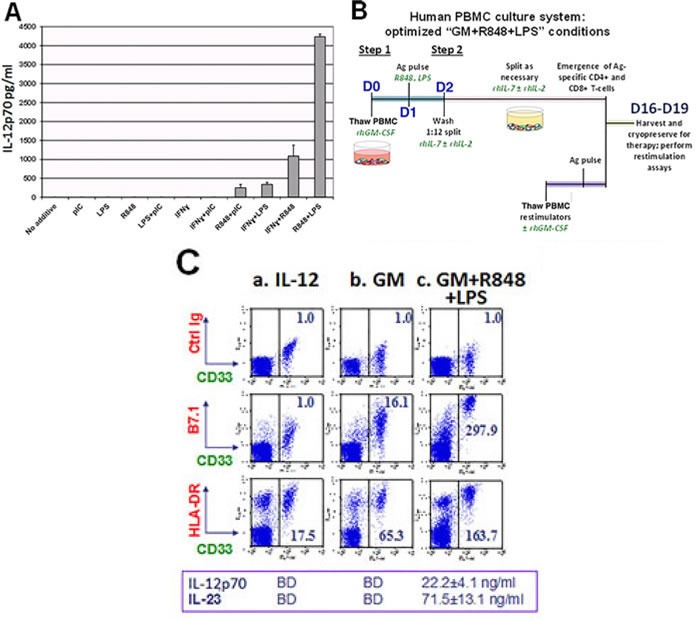
Unfractionated PBMC contain highly serviceable, readily activated myeloid Ag-presenting cells **A**. Cryopreserved healthy donor PBMC derived by leukapheresis were thawed on d0, cultured overnight in RPMI 1640 with standard additives,10% heat deactivated human AB serum, 40ng/ml rhGM-CSF and 20ng/ml rhIL-4, then exposed on d1 to single or paired danger signals (pIC 50μg/ml, R848 10μM, LPS 50ng/ml, IFNγ 2000 IU/ml). Supernatants were harvested for ELISA on d2 to determine production of IL-12 (functionally assembled IL-12p70 dimer), pg/ml, 6 million PBMC/ml. This is representative of 8 biological replicates. **B**. Scheme depicting Steps 1 and 2 of culture for groups receiving rhGM-CSF+R848+LPS. **C**. d2 analyses of PBMC cultured three different ways: (a) standard “IL-12” culture group (2 days prior to their initial exposure to rhIL-12 and rhIL-2); (b) “GM” conditioned culture group which received rhGM-CSF (40 ng/ml) on d0; (c) “GM+R848+LPS” group, same as “GM” group but also receiving R848+LPS on d1. d2 supernatants were assayed for IL-12p70 and IL-23 (BD = below detection) and resuspended PBMC were analyzed by FACS for B7.1 and HLA-DR expression within the CD33+ myeloid subpopulation. Mean fluorescent indices (MFI) were normalized relative to isotype control values of 1.0, and are representative of 5 biological replicates. **D**. Same groups as in **C**., carried forward in culture after pulsation either with Candida albicans extract at a 1:10 dilution (CAN) or with recombinant HER2 intracellular domain protein (HER2-ICD 50 μg/ml). “IL-12” groups were Ag-pulsed d0 prior to receiving rhIL-2 and rhIL-12 on days 4, 8 and 12 (60 IU/ml and 10 ng/ml respectively). “GM” and “GM+R858+LPS” groups received GM d0, were Ag-pulsed d1 with or without R848+LPS, and transitioned to IL-7/IL-2 treatment d2. All groups were harvested on d16 (end of “Round One”), and tested in an ICC assay for specific reactivity to freshly thawed Ag-pulsed autologous PBMC. The “GM+R848+LPS” groups were also carried forward for Round Two of culture. Restimulator PBMC were either unpulsed (UP), or pulsed with CAN or HER2-ICD, and added at a T-cell:restimulatory PBMC ratio of 2:1. Fold expansion is indicated for each group. Shown dot plots are gated on the CD4+ subpopulation, with CD8+ T-cell responses displaying a parallel trend (not shown). Numbers in each dot plot show each culture condition's frequency of IFNγ+ CD4+ T-cells, calculated as %RUQ/(%RUQ+%RLQ). Representative of two biological replicates.

In preliminary experiments, previously cryopreserved, freshly thawed unfractionated PBMC from unvaccinated healthy volunteers were exposed for one day to conventional DC differentiation stimuli (recombinant human (rh) GM-CSF (GM) and rhIL-4), then overnight to a variety of innate immunity stimuli, after which ELISA was performed on culture supernatants to measure PBMC secretion of IL-12p70 (assembled IL-12 dimer). As shown in Figure 1A and Supplemental Figure S1A, a range of responses was observed among individual donors and culture media, but the paired addition of the TLR4 agonist LPS and the TLR8 agonist resiquimod (R848) was by far the strongest and most consistent rapid inducer of IL-12p70 production. Furthermore, exposure to GM and/or IL-4 prior to R848+LPS was itself responsible for a log fold augmentation of IL-12p70 secretion (Supplemental Figure S1B). Intracellular IL-12p70 assays confirmed that IL-12 production was attributable to the CD33+ myeloid fraction of PBMC (Supplemental Figure S1C). Consistent with the absence of TLR9 and TLR7 expression on human myeloid cells [25], CpG and imiquimod proved ineffective for inducing IL-12p70 production (data not shown).

Even though initial conditioning of human PBMC with either IL-4 or GM logarithmically enhanced R848+LPS induced IL-12p70 production (Supplemental Figure S1B), preliminary experiments revealed that initial conditioning with GM alone (omitting IL-4) was most effective for culture-expanding IFNγ-producing, Ag-specific T-cells *in vitro* (data not shown). We therefore directly compared the T-cell sensitizing impacts of standard culture in exogenous IL-12 (“IL-12”) to upfront conditioning with exogenous GM (“GM”) or GM plus R848 and LPS (“GM+R848+LPS”), when PBMC from healthy unvaccinated donors were pulsed with either Candida albicans extract (CAN) or with recombinant HER2 intracellular domain protein (HER2-ICD) (Figure 1B illustrates conditions for the “GM+R848+LPS” groups).

Phenotypic analyses and ELISAs of IL-12 and IL-23 production were performed on d2 of cultures. At that juncture, the “IL-12” group had not yet received exogenous IL-12 or IL-2, and was notable for modest major histocompatibility complex (HLA-DR) expression, an absence of co-stimulatory B7.1 (CD80) within the CD33+ (myeloid) fraction, and undetectable endogenous IL-12p70 or IL-23 secretion. By comparison, exposure to exogenous GM beginning on d0 increased expression of both HLA-DR and B7.1 expression within the CD33+ subpopulation without inducing IL-12p70 or IL-23 secretion, whereas exposure both to GM on d0 and to R848+LPS on d1 (GM+R848+LPS) further matured the CD33+ subset and also stimulated prodigious IL-12p70 and IL-23 secretion (Figure 1C). In addition, expression of the alpha X-chain integrin CD11c, normally restricted to the small subpopulation of precommitted DC within the CD33+ PBMC fraction [30, 31], extended to the majority of CD33+ cells after exposure to GM and/or R848+LPS (Supplemental Figure S1D).

The “GM” and “GM+R848+LPS” culture groups were expanded from d2 forward in exogenous IL-7+IL-2 (Figure 1B) whereas standard “IL-12” groups received IL-12 and IL-2 on days 4, 8 and 12. Intracellular cytokine assays (ICC’s) were performed at d16 of culture. Compared to exogenous IL-12 or GM conditioning, the combination of GM+R848+LPS markedly increased both the absolute numbers and the frequency of either CAN- or HER2-ICD-specific, IFNγ-producing T-cells, both CD4+ and CD8+ (Figure 1D and not shown). This was already observable within a single 16-19 day round of culture, and was easily amplified further by a second round of Ag-driven culture (Figure 1D).

### IL-7 uniquely extends the proliferation and survival of GM+R848+LPS conditioned, Ag-specific CD4+ and CD8+ T-cells from unfractionated PBMC

**Figure 2:**
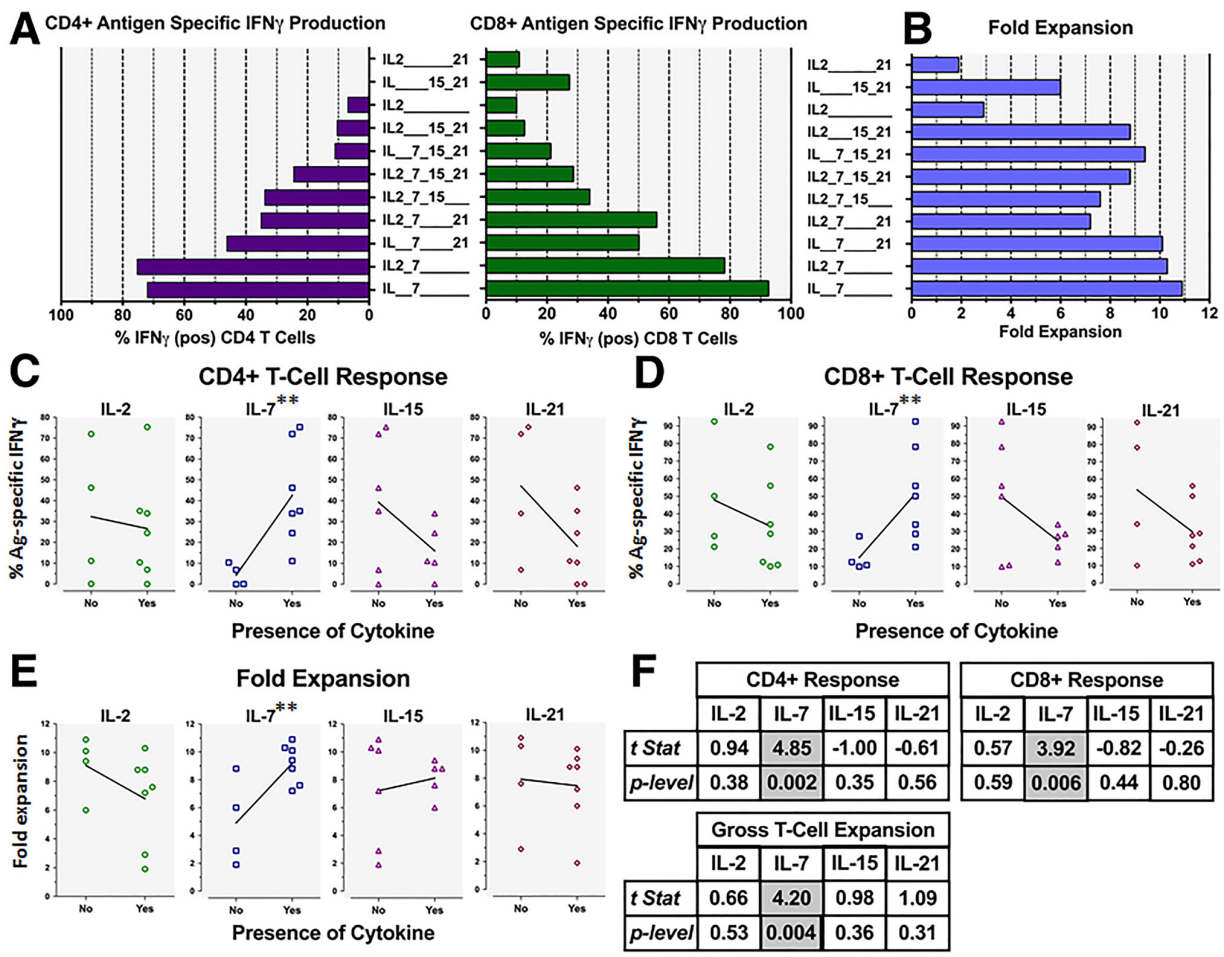
γ-chain cytokines have varying impacts on GM+R848+LPS modulated Ag-specific PBMC T-cell expansion Cultures as in Figure 1D, but all groups received GM, CAN, R848 and LPS prior to exposure to various γ-chain cytokines from d2 to d16 (see doses under Materials and Methods). Graphs depict ICC analyses of harvested T-cells on d16, namely: **A**., left panel, frequency of CAN-specific IFNγ+ CD4+ T-cells; **A**., right panel, frequency of CAN-specific IFNγ+ CD8+ T-cells; and **B**., gross fold culture expansion. **C**./**D**./**E**. Linear regression analyses comparing the presence (“NO *vs* YES”) of each cytokine, indicating that among the γ-chain cytokines tested (rhIL-7, rhIL-2, rhIL-15 and rhIL-21), only rhIL-7 had a significantly positive association with T-cell specificity, both CD4+ and CD8+, as well as with yield. Representative of two biological replicates. See **F**. and Supplemental Table S1 for details of the statistical analysis.

We examined the impact of different γ-chain cytokines, individually or in combination, upon the expansion of Ag-specific CD4+ or CD8+ T-cells from unfractionated, GM+R848+LPS conditioned PBMC. Previous reports have observed that IL-7 and IL-21, IL-7 and IL-15 or IL-15 and IL-21 can synergize to enhance generation of Ag-specific T cells [32–36]. Surprisingly, for GM+R848+LPS conditioned PBMC, rhIL-7 or rhIL-7+rhIL-2 proved far superior to other γ-chain cytokines, whether singly or in combination, for licensing selective expansion and/or survival of both CD4+ and CD8+ Ag-specific T-cells during 2-3 week culture (Figure 2A left and right panels). Furthermore, co-exposure to any examined γ-chain cytokine(s) other than IL-2 reduced IL-7's ability to license Ag-specific T-cell expansion (Figure 2A, 2B). Multiple linear regression analyses (Supplemental Table S1 and Figure 2F) revealed that only IL-7 exposure was significantly associated with CD4+ T-cell Ag-specificity (Figure 2A, 2C), CD8+ T-cell Ag-specificity (Figure 2A, 2D), and fold expansion (Figure 2B, 2E) (*p* = 0.00185**, 0.00406**, and 0.00572** respectively).Thus, while GM+R848+LPS conditioning could license preferential expansion of Ag-driven PBMC T-cells, exposure to rhIL-7 proved to be an equally critical co-licensing requirement for maximally sustained Ag-specific proliferation and survival.

### CD4+ and CD8+ T-cells with enriched specificity for tumor-associated long synthetic peptides are readily expanded from GM+R848+LPS conditioned, unfractionated PBMC

**Figure 3:**
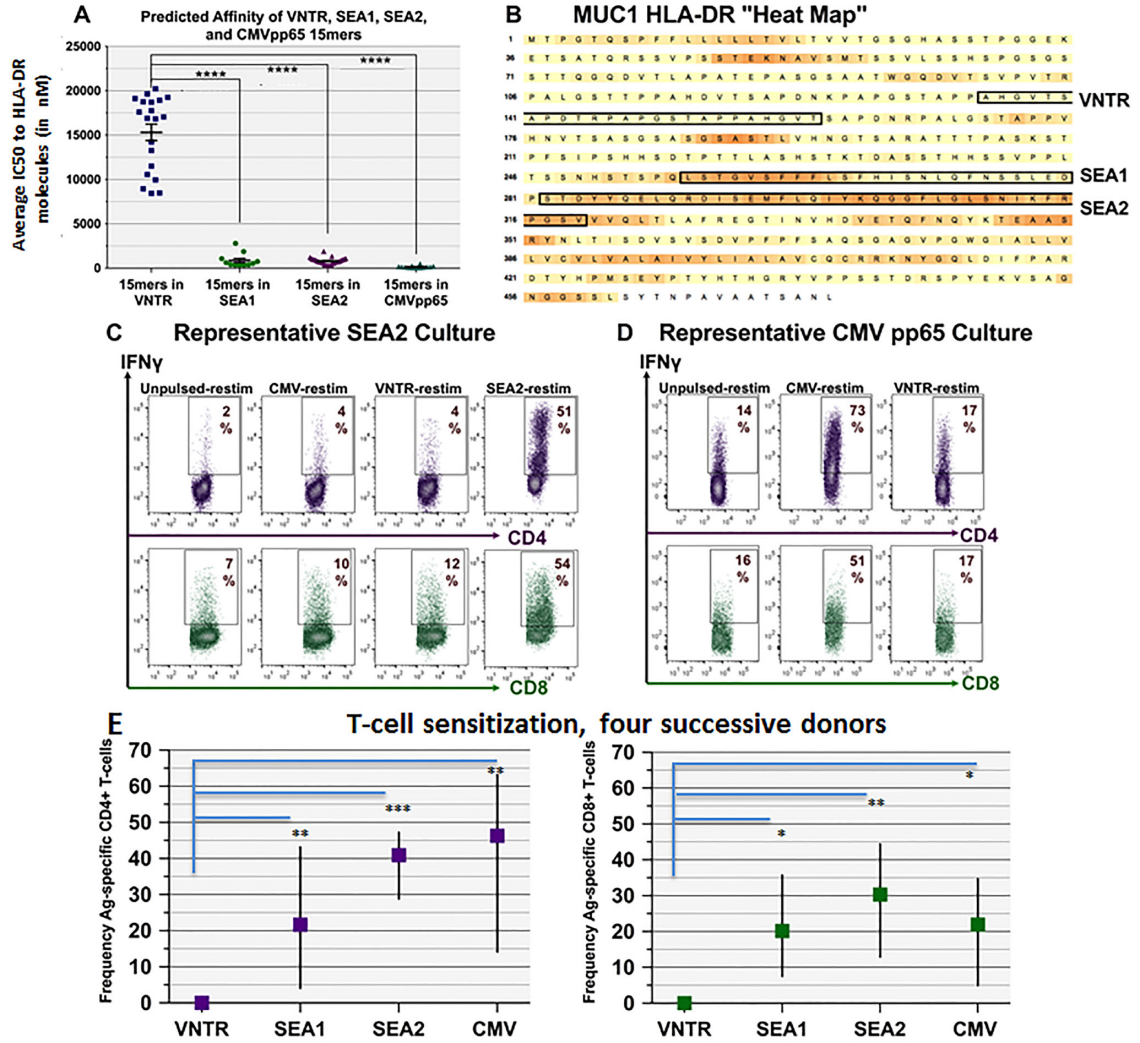
CD4+ and CD8+ natural T-cells recognizing MUC1- and CMV-associated peptide sequences can be reliably propagated even from non-vaccinated PBMC donors Utilizing known HLA-DR frequencies, a composite array of 29 HLA-DR haplotypes was identified that together spanned nearly 90% of individuals regardless of race or ethnicity (Supplemental Table S2). Open access Ag discovery software (NetMHCIIpan 2.1-3.0 and NetMHCpan 2.4-2.8) was employed to identify “hot spot” domains within individual tumor-associated proteins containing clusters of embedded 15mers with promiscuously high affinity for the composite HLA-DR array, as well as embedded 8-9mers predicted to have high affinity for the widely prevalent HLA-A2.1 haplotype. **A**. Predicted averaged HLA-DR binding affinities for all 15mers contained within three MUC1 domains (VNTR, SEA1 and SEA2) as well as CMVpp65. Data are portrayed as IC50 in nM, with lower values indicating higher affinity. Affinities for SEA1- and SEA2-derived 15mers were observed to be significantly stronger than VNTR-derived 15mers (two-tailed *p* always < 0.0001****). **B**. A heat map was derived for MUC1 showing a relative dearth of hot spots compared to HER2/neu (Figure 4A) or to CMV pp65 protein (not shown). **C**.-**D**. Representative ICC outcomes when PBMC were cultured with GM+R848+LPS, pulsed with SEA2 or CMVpp65 (50μg/ml), then expanded in rhIL-7 to d16. Restimulation PBMC were either unpulsed or pulsed with CMVpp65, SEA2, or VNTR peptide. Dot plots are gated on CD4+ or CD8+ T-cells. Both CD4+ and CD8+ IFNγ-producing T-cells were readily sensitized and expanded. **E**. Graphic summary for 4 successive healthy unvaccinated PBMC donors, vertical lines showing the range of responses (frequency of Ag-specific, IFNγ-producing T-cells) and the boxes showing averaged response of the 4 donors. Culture succeeded in expanding T1-type CD4+ and CD8+ T-cells recognizing SEA1, SEA2 and CMVpp65 from all 4 donors, but not VNTR (latter significantly different compared to the other three Ags with two-tailed *p* ranging from 0.03* to 0.0002***; no significant differences among the other three Ags).

**Figure 4:**
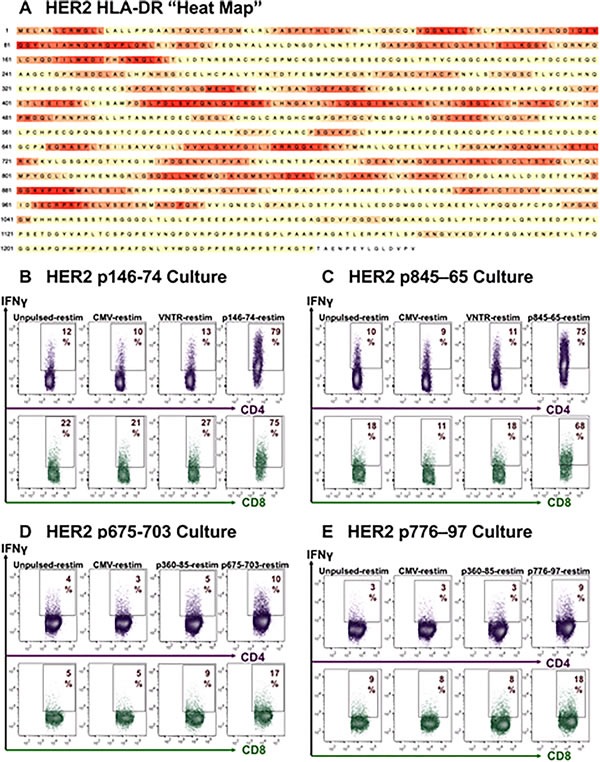
HER2/neu specific natural CD4+ and CD8+ T-cells are readily propagated in culture **A**. Heat map of HER2/neu derived as described in Figure 3. In contrast to MUC1, “hot spots” are numerous. **B**./**C**./**D**./**E**. d16 ICC's showing natural sensitization of both CD4+ and CD8+ PBMC T-cells driven by 4 different HER2/neu-derived peptide sequences, with specific reactivity apparent for the sensitizing peptides, but not to CMVpp65, VNTR or to other HER2-derived peptides absent during the prior sensitization culture.

Because it is often difficult and/or costly to prepare clinically acceptable grades of complex proteins for use as Ags, we investigated whether GM+R848+LPS conditioned PBMC could process exogenous synthetic peptides as effectively as CAN extract or recombinantly produced HER2-ICD. We therefore sought to identify long synthetic peptides (20mers or longer) that had the potential to be truly universal HLA-DR (MHC class II) binders, based on cleavable embedded 15mer peptide sequences [37], as well as cleavable embedded 8-9mers with high avidity for the widely prevalent MHC Class I haplotype HLA-A2.1 (see Materials and Methods), We applied this strategy to predict immunogenic “hot spots” within the widely prevalent tumor-associated proteins MUC1 and HER2 (Figure 3 and Figure 4), as well as the brain tumor-associated pp65 cytomegalovirus (CMV) protein [21, 38–41].

Employing this algorithm, we identified and synthesized leading candidate peptide sequences from MUC1, HER2 and CMVpp65. Predictions encompassed both previously identified [42–45] and heretofore unidentified epitopes. Compared to HER2, which was abundant in hot spot regions (Figure 4A), MUC1 displayed few hot spots, with the leading MUC1 candidate sequences proving to be within the Sperm protein, Enterokinase and Agrin (SEA) domain, designated SEA1 and SEA2 (Figure 3A, 3B). Ironically, the most widely studied MUC1 region, the variable number of tandem repeats (VNTR) domain [46], was predicted to be much less effective as an immunogen, at least in a nonglycosylated state (Figure 3A, difference in composite IC50 for VNTR *versus* SEA1, SEA2 or CMVpp65 *p* < 0.0001****).

The synthesized long peptides were individually pulsed onto GM+R848+LPS conditioned PBMC cultures established from unvaccinated HLA-A2.1+ healthy volunteers, then expanded in rhIL-7 or rhIL-7+rhIL-2. Within 16 culture days it was consistently possible to numerically expand and significantly increase the frequency of T-cells with natural specificity for MUC1, HER2, or CMVpp65, both CD4+ and CD8+ (Figure 3 and Figure 4). As predicted from our algorithm, the SEA1 and SEA2 MUC1 sequences as well as CMVpp65 were highly immunogenic, whereas the VNTR non-glycosylated sequence was reproducibly less effective (Figure 3E). Proliferation of CD4+ and CD8+ T-cells was indistinguishable, with both subsets retaining their initial proportionality during expansion (data not shown).

HER2-specific CD4+ and CD8+ T-cell responses could also be readily achieved against tested long peptides, some of which contained embedded promiscuous15mers that we have previously described and utilized in clinical trials [23, 24, 43] (Figure 4B/4C/4D/4E).

### Phenotypic features of Ag-specific T-cells derived during GM+R848+LPS stimulated human PBMC cultures

**Figure 5:**
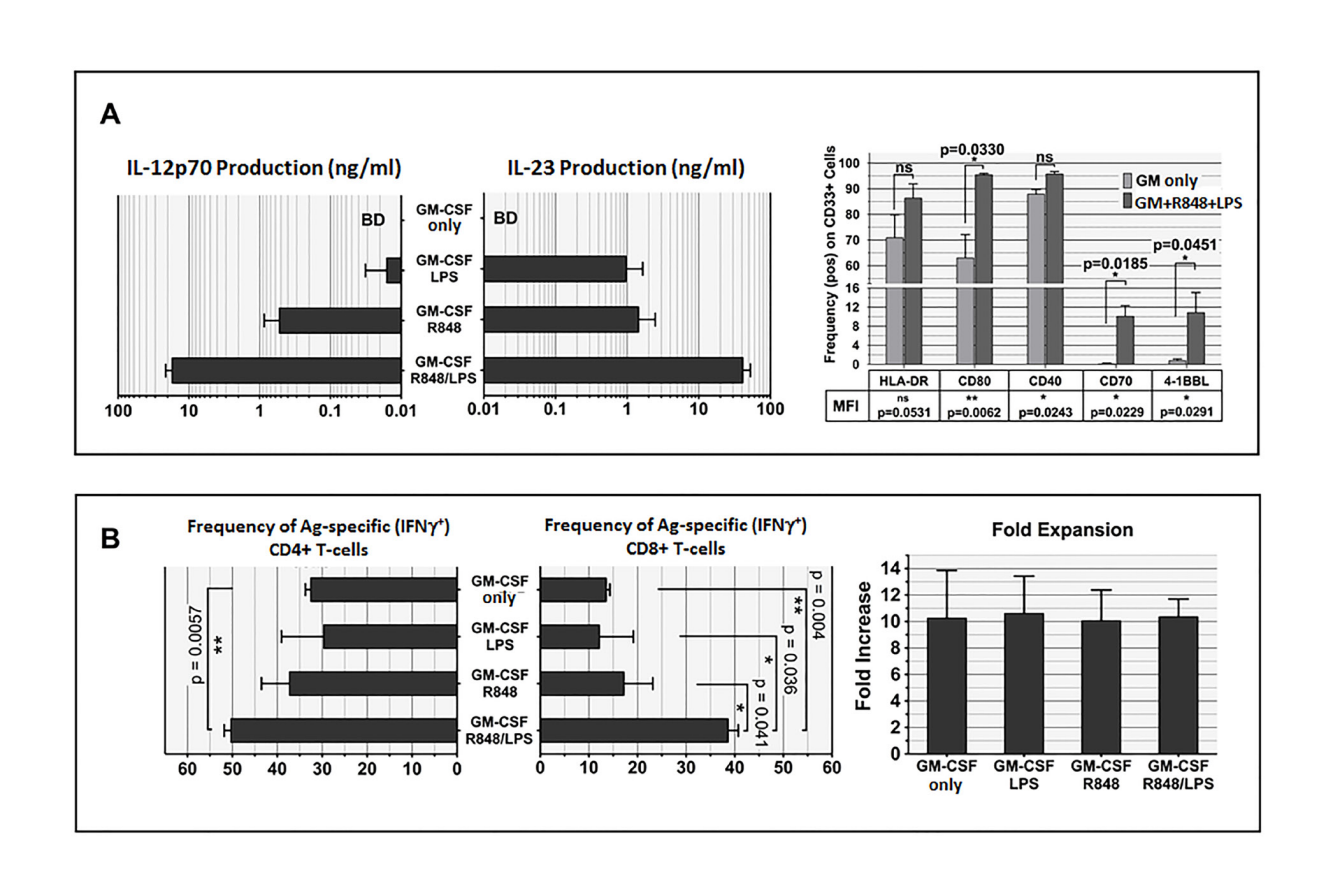
Ag-specific T1 cytokine and lytic profile dominate outcomes of GM+R848+LPS+IL-7 conditioned, Ag-driven cultures Cultures were conducted as in Figure 3 and Figure 4. **A1**. Comprehensive representative Luminex cytokine profile when a donor's T-cell culture was Ag-driven by either CMVpp65 or SEA2 peptide, then restimulated overnight on d16 with unpulsed PBMC *vs* PBMC pulsed with the driving peptide (e.g., SEA2♢UP *vs* SEA2♢SEA2). **A2**. Meta-analysis of Luminex assays including five biological replicates per cytokine (three cultures of SEA2-driven T-cells and 2 cultures of CMVpp65-driven T-cells), showing averaged Ag-specific cytokine release in log scale. IFNγ production exceeded that of any other cytokine by one to three logs, a difference which was significant (two-tailed *p* range from 0.0240* to 0.0158*). **B**. Capacity of T-cells from cultures driven by SEA1 or SEA2 peptides to preferentially lyse MUC1-expressing tumor targets. HLA-A2.1+ PBMC from 3 healthy donors were either driven polyclonally with anti-CD3/CD28 (see Methods) or driven by long peptides synthesized from the SEA domain of MUC1 (SEA1 and SEA2) with GM+R848+LPS+IL-7 conditioning. At the end of T-cell culture expansion, each of the T-cell groups was cultured for 8h at a 100:1 ratio with Cr^51^-labelled HLA-A2.1+ human breast cancer line MDA-MB-231, either transduced to express MUC1 (MDA-MB-231.MUC1) or Neo control (MDA-MB-231.Neo). % Lysis was calculated as ((Experimental Lysis - Spontaneous Cr^51^ release)/(Complete Lysis in Triton X-100 - Spontaneous Cr^51^ release)) x 100. % lysis of MDA-MB-231.Neo was statistically indistinguishable for all 3 donors whether cultures were polyclonally-, SEA1- or SEA2-driven. Polyclonally driven T-cells from all 3 donors lysed MDA-MB-231.MUC1 indistinguishably from MDA-MB-231.Neo. In contrast, SEA1- and SEA2- driven T-cells from all 3 donors lysed MDA-MB-231.MUC1 targets significantly more than MDA-MB-231.Neo targets (two-tailed *p* = 0.039 and 0.038 applying Student's paired *t*-test).

GM+R848+LPS conditioned, IL-7 or IL-7+IL-2 expanded PBMC T-cells were analyzed for their abilities to produce Ag-specific cytokines in restimulatory Luminex assays of supernatants as well as in restimulatory ICCs. Although a mixture of T1-, T2- and T17-type cytokines were produced by the bulk cultures, a predominantly T1-type IFNγ-producing response was evident in the Luminex assays (Figure 5A1, 5A2), paralleling ICC results (e.g., Figure 3C, 3D). Furthermore, T-cells from GM+R848+LPS conditioned PBMC driven by either SEA2 or SEA1 long peptides not only specifically recognized one or both of these sensitizing peptides at restimulation, but also preferentially lysed the HLA-A2.1+ human breast cancer line MDA-MB-231 transduced to express MUC1 (Figure 5B).

Subanalysis of CD4+T-cells producing Ag-specific IFNγ demonstrated nearly uniform expression of the B7.1 costimulatory receptor CD28, as well as a near absence of CD56, consistent with a favorably multipotent, non exhausted state of T-cell differentiation (Supplemental Figure S2A [47–49]). Furthermore, a mix of CCR7+ and CCR7- T-cells was expanded in culture, consistent with dual maintenance of central memory and memory effector T-cells (Supplemental Figure S2A [50, 51]). Co-staining in ICC's for multiple intracellular cytokines was also performed to see if individual Th1 and Th17 subpopulations were discernable. In such experiments, we observed that individual T-cells produced every possible combination of IFNγ, IL-17 and IL-2 upon reexposure to their driving Ag (Supplemental Figure S2B).

We investigated whether regulatory T-cells (Tregs) were conspicuous in these cultures. Although a subset of T-cells expressed Foxp3 at culture's end (Supplemental Figure S3), this subset was almost completely lacking co-expression of latency-associated peptide (LAP) and/or glycoprotein A repetitions predominant LRRC32 (GARP) (Supplemental Figure S4), a composite phenotype associated with activated effector T-cells rather than with Treg function [52–54]. A parallel absence of LAP and/or GARP expression was also observed for Helios+ T-cells (Supplemental Figure S4), indicating that they too were not Tregs [53, 54]. Finally, as expected, subsets of both CD4+ and CD8+ T-cells displayed physiologic checkpoint receptors for B7.1 (CTLA4) and/or for PD-L1 (PD-1) (Supplemental Figure S3 [55, 56]).

Preliminary PBMC cultures established from four breast cancer patients indicated that the presently described culture system could be applied successfully to patients with malignancies (example shown in Supplemental Figure S5A). Furthermore, cocktails of long peptides could be employed to simultaneously expand T-cells encompassing a variety of oncoprotein specificities (Supplemental Figure S5A and S5B). PBMC cultures could also be conducted in expansile 1 liter vessels (G-Rex 100M [57]) at least as effectively as in 24 well cluster plates (Supplemental Figure S5B). Finally, T-cells cryopreserved at the end of culture fully retained their capacity for Ag-specific IFNγ production upon subsequent rethaw (data not shown).

### R848 and LPS together produce maximal expansion of Ag-specific, IFNγ-producing CD4+ and CD8+T-cells from PBMC

**Figure 6:**
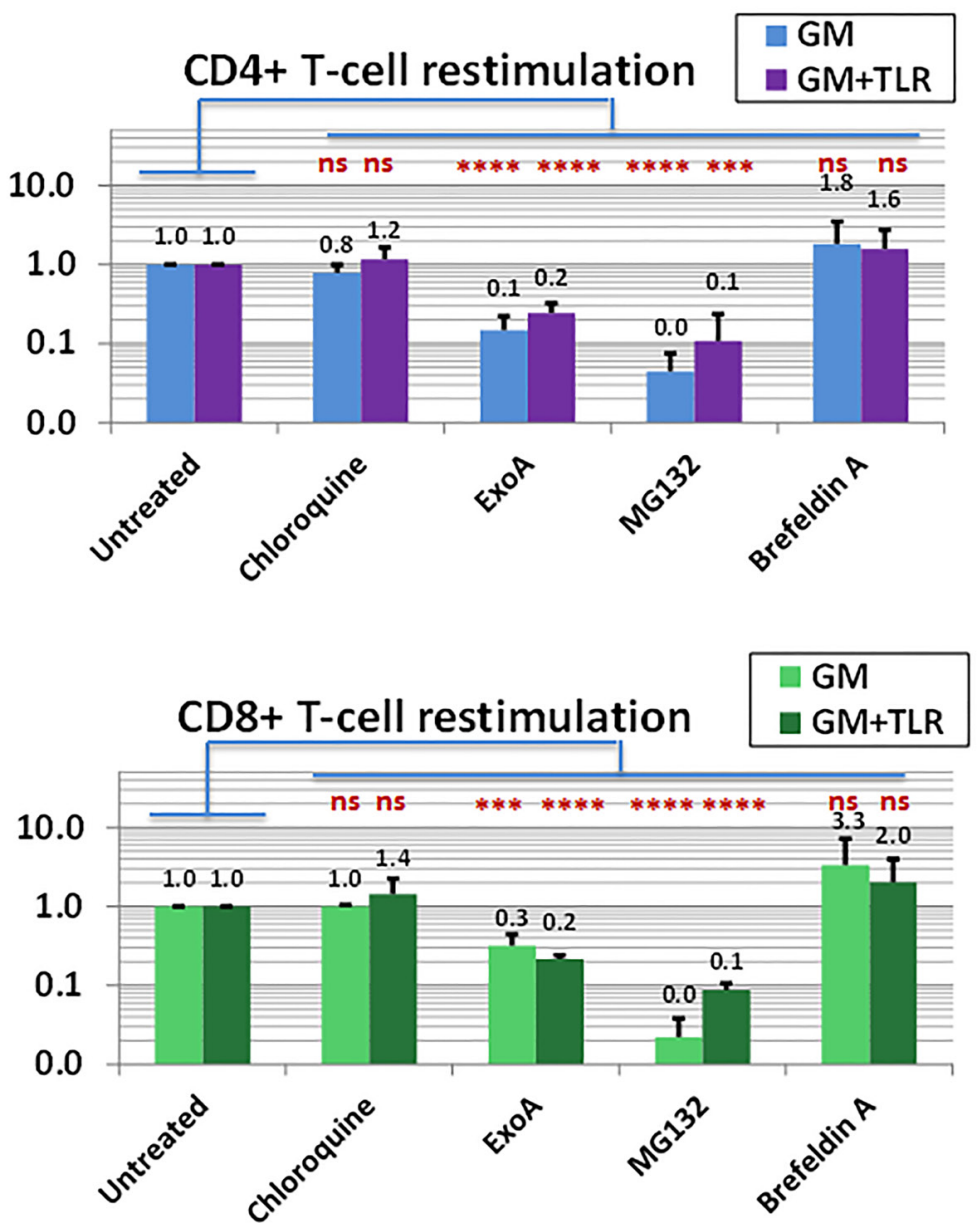
Exposure to R848 and LPS is a critical determinant of CD8+ T-cell sensitization **A**. left panel. IL-12 and IL-23 production during first two days of culture are both markedly enhanced by dual stimulation with R848 and LPS. Methods as in Figure **1B**. Note that cytokine production is shown in log scale. This graphing of ELISA results shows an averaging of 4 biological replicates. **A**. right panel, exposure to R848+LPS enhances frequency and/or density of co-stimulatory ligands and receptors on PBMC CD33+ constituents. Methods as in Figure **1B**., with FACS analyses performed after 48h in culture. Frequency of each molecule of interest in the CD33+ subpopulation was determined and averaged for 4 biological replicates; in addition, fold-increase in cell surface staining density relative to isotype control (averaged Mean Fluorescent Index) was determined. Frequency of expression is shown graphically, whereas for MFI only tabulated *p* values are shown. Exposure to R848+LPS significantly increased both the frequency and MFI of CD70, 4-1BB ligand, and B7.1 (CD80) (shown on graph). In addition, although frequency of CD40 expression was already nearly uniform without R848+LPS exposure, R848+LPS significantly increased the MFI for CD40. Two-tailed *p* values are shown, based on a paired Student *t*-test comparing with and without R848+LPS exposure within each experimental run. B, left panel, dual exposure to R848+LPS significantly enhances Ag-specific CD4+ and CD8+ propagation. Cultures were initially pulsed with CAN, with or without co-exposure to R848 and LPS, performing an ICC assay at the end of culture to enumerate frequency of CAN-specific, IFNγ-producing CD4+ and CD8+ T-cells. Graphs show an averaging of 2 biological replicates. Two-tailed *p* values are shown, based on a paired Student *t*-test comparing with and without R848 and/or LPS exposure within each experimental run. B, right panel, averaged gross numeric expansions of all cells for each culture condition, corresponding to experimental runs in the left panel, demonstrating that gross numeric expansion *per se* is not predictive for enrichment of T-cell specificity.

To understand the mechanism(s) behind the enhanced generation of Ag-driven specific T-cells observed with GM+R848+LPS conditioning, we performed further analyses on d2 cultures, 24h after Ag-addition. Conditioning with GM alone was unable to induce detectible IL-12 or IL-23 secretion (Figure 6A left panel). In contrast, GM+R848 conditioning (omitting LPS) resulted in moderately potent production of IL-23 and IL-12, and GM+LPS (omitting R848) conditioning resulted in IL-23 but not IL-12 production. Complete GM+R848+LPS conditioning maximally raised both IL-12 and IL-23 secretion (Figure 6A left panel).

We examined the expression of co-stimulatory and other molecules in the CD33+ myeloid fraction of PBMC as a consequence of R848+LPS exposure (Figure 6A right panel). We examined changes in both the frequency of expression (shown graphically) and the intensity of expression (data not shown except for *p* values). Compared to conditioning with GM alone, GM+R848+LPS conditioning significantly licensed *de novo* expression, both frequency and intensity, of the costimulatory ligands CD70 and 4-1BBL within the CD33+ myeloid subpopulation (Figure 6A right panel). Both of these ligands are strongly associated with the promotion and sustenance of Ag-driven CD8+ T-cell responses [58–63]. R848+LPS exposure also triggered a significantly higher frequency and more intense expression of the co-stimulatory molecule B7.1 (CD80) (Figure 6A right panel). R848+LPS exposure did not enhance the frequency of CD40 expression, which was already nearly uniform with GM alone, but R848+LPS exposure significantly increased the intensity of CD40 expression, potentially enhancing CD40 ligand-mediated licensure of crosspresentation to CD8+ T-cells [64] (Figure 6A right panel).Taken as a whole, GM+R848+LPS strongly biased the CD33+ myeloid subset of PBMC to acquire the characteristics of DC1-polarized, IL-12-secreting, fully matured DC [25].

Next, we examined the individual impacts of R848 and/or LPS upon subsequent Ag-specific T-cell generation during exposure to IL-7+IL-2. GM conditioning with omission of R848 and/or LPS more severely limited the expansion of Ag-specific CD8+ T-cells than of CD4+ T-cells, indicating that reliable MHC Class I cross-presentation of exogenous Ag was particularly enhanced by aggressive activation of innate immunity (Figure 6B, left and mid panel). Notably, in contrast to Ag-specific frequency, gross numerical cell expansion was not significantly different across all the GM-conditioned treatment groups (Figure 6B right panel), indicating that supplementation of GM with R848+LPS enhanced selective proliferation and/or preferential survival of Ag-driven T-cells over bystander T-cells [65].

Given the diverse impacts of GM+R848+LPS upon the CD33+ PBMC subpopulation, we sought to determine whether the observed enhancements of both CD4+ and CD8+ T-cell Ag-driven propagation were absolutely dependent upon the CD33+ subpopulation's production of IL-12 and/or IL-23. Quantitative mAb neutralization of IL-12 and/or IL-23 during GM+R848+LPS cultures proved to have no significant impact, either deleterious or enhancing, upon propagation of Ag-specific, IFNγ-producing CD4+ and CD8+ T-cells (data not shown), indicating that composite effects of GM+R848+LPS upon DC1 polarization were sufficiently diverse to reduce dependence upon any individual factor such as IL-12.

### PBMC-based Ag-presenting cells avoid acidification and utilize the proteasome and Sec61 for both MHC Class I and II presentation of exogenous Ag

To delineate the pathway(s) involved in PBMC-based Ag-processing and presentation, we performed restimulation ICC assays in which freshly thawed autologous PBMC were conditioned with GM or GM+R848+LPS, Ag-pulsed, and concurrently exposed to known inhibitors of Ag-processing, before being extensively washed and introduced as restimulators for Ag-driven T-cells after 16 days’ culture.

Under the classical pathway of exogenous Ag-processing, Ag is internalized in endosomes which fuse with acidifying lysosomes for purposes of Ag trimming. For MHC Class I cross-presentation of exogenous Ag, which rarely occurs spontaneously, Ags retrotranslocate into the cytosol from the late endosome for proteasome-mediated processing, followed by Transporter Associated with Ag Processing (TAP)-mediated importation into the endoplasmic reticulum (ER), loading onto MHC Class I molecules, transfer to the Golgi apparatus, and export to the cell surface [66, 67]. In the case of MHC Class II, endosomes fuse with vesicles exported by the Golgi apparatus containing MHC Class II and invariant chain to become the late endosomal MHC class II compartment, which loads peptide onto MHC Class II complexes for transport to the cell surface [66, 68].

**Figure 7:**
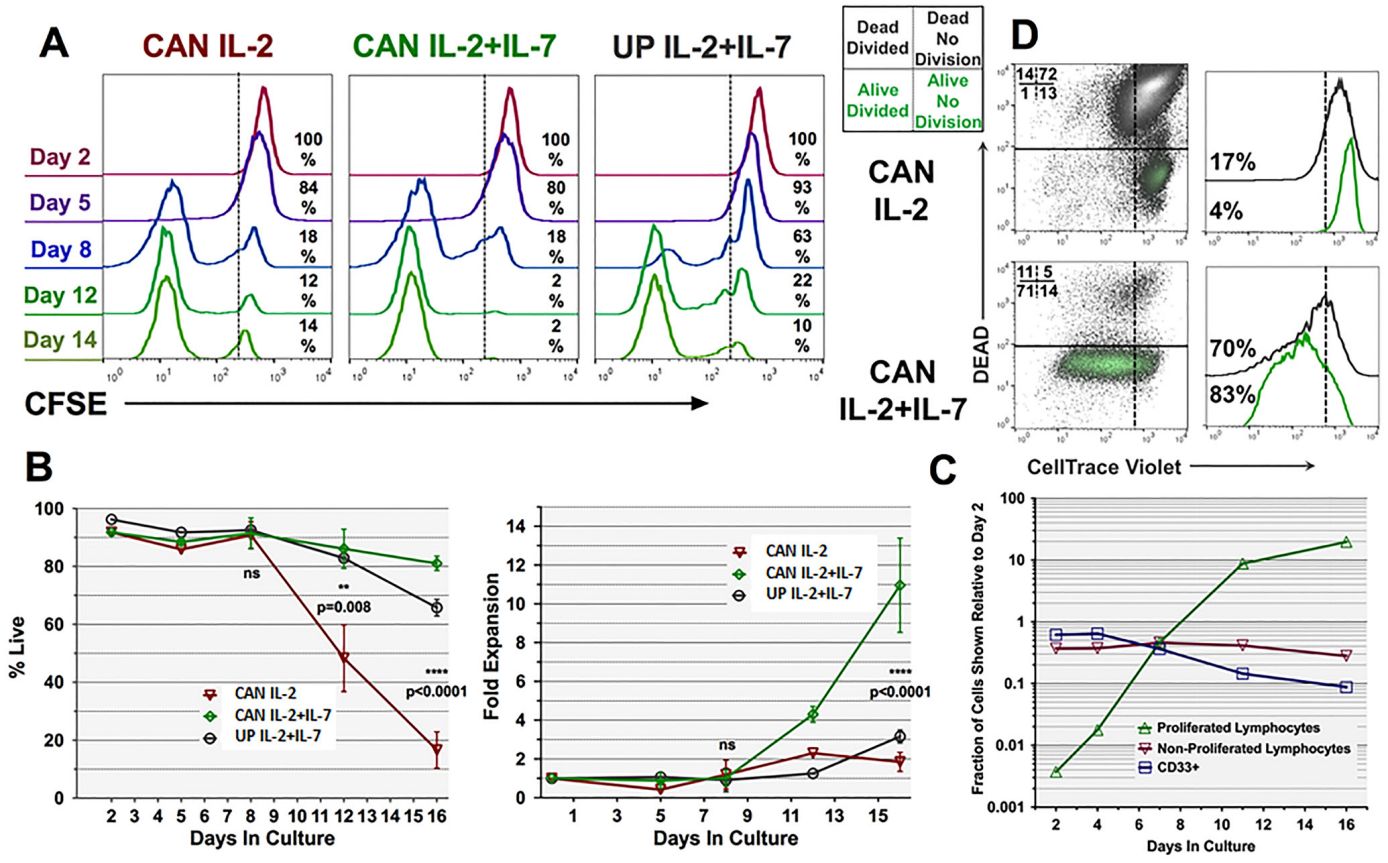
APC within the PBMC compartment forego acidification of exogenous Ag and utilize the proteasome and Sec61 for both MHC Class I and II presentation Freshly thawed PBMC were activated with GM alone or GM+R848+LPS; unpulsed or pulsed with SEA1, SEA2 or CMVpp65 peptides; and concurrently exposed to standard doses of individual inhibitors of Ag processing: the acidification inhibitor chloroquine (CQ, 100μM), the Sec61 transport inhibitor ExoA (100 ng/ml), the proteasome inhibitor MG132 (10μM) and the ER/Golgi transport inhibitor Brefeldin A (BFA, 10 μg/ml). At 48h each group was harvested, washed thoroughly to remove inhibitors, confirmed for intact viability, and used to restimulate autologous d16 T-cell cultures previously expanded to specifically recognize.SEA1, SEA2, or CMVpp65. The ICC assay measured, as a readout for Ag-presentation, the frequency (%) of cultured CD4+ and CD8+ T-cells achieving Ag-specific IFNγ production when exposed to restimulatory PBMC pulsed with the relevant sensitizing peptide (minus the background frequency of IFNγ+ T-cells exposed to unpulsed restimulatory PBMC). Readouts were normalized relative to the groups receiving restimulatory PBMC not treated with inhibitors, which were scored as 1.0. Each bar represents the average ±SD of three determinations under the same conditions, one for each driving peptide, with the impacts of the inhibitors not proving significantly different for GM *vs* GM+R848+LPS conditioning, There was also no significant difference between “No inhibitor” groups and groups receiving CQ or BFA, whereas “No inhibitor” *vs* ExoA and “No inhibitor” *vs* MG132 were highly significant (two-tailed *p* consistently < 0.0007*** for both CD4+ and CD8+ T-cells). Results testing another proteasome inhibitor, lactacystin, closely paralleled the impacts observed with MG132 (data not shown).

Downstream disruption of classical Ag-processing pathways can be accomplished by treatment with brefeldin A (BFA), an inhibitor of ER-Golgi transport that blocks ER export of both MHC Class I and Class II molecules [69]. Surprisingly, when 16d T-cell cultures were tested in restimulation ICC assays, treatment of the freshly prepared restimulatory PBMC with BFA did not significantly abrogate Ag-specific responses, and often paradoxically enhanced them (Figure 7). BFA's lack of an inhibitory effect was observed both for GM and for GM+R848+LPS conditioning, and occurred whether the restimulatory Ag was SEA1, SEA2 or CMVpp65.

These results indicated that an alternative Ag-processing mechanism(s) able to bypass the ER/Golgi was operative [68]. One such alternative pathway is a variant of the ER-associated degradation (ERAD) pathway in which endosomes themselves acquire features more typically associated with ER, such as MHC-Class I, TAP, Sec61, and p97 expression. This allows exogenous Ags in professional Ag-presenting cells (APC) to bypass ER/Golgi-dependent Ag processing, helpfully segregating the processing of exogenous from endogenous Ags [70–73]. Best studied for MHC Class I-restricted responses, Sec61 and p97 translocate exogenous Ags from endosomes into the cytosol, where they are processed by the proteasome, before reentering endosomes for loading onto MHC Class I complexes.

To test the potential role of an ERAD-like pathway in GM+R848+LPS conditioned PBMC cultures, we utilized MG132 and lactacystin (LT), both well-characterized proteasome inhibitors [74], and *Pseudomonas aeruginosa* Exotoxin A (ExoA), an inhibitor of Sec61-mediated Ag translocation previously shown to diminish endosome-dependent processing [72, 73]. Even though MG132, LT and ExoA exerted no detectable toxic effects upon restimulator PBMC, treatment with any of these three agents significantly diminished the restimulators’ ability to trigger both CD8+ and CD4+ T-cell Ag-specific IFNγ production (*p* range from 0.0001**** to 0.0006***). This was observed equally for GM and for GM+R848+LPS conditioning, and occurred whether the restimulatory Ag was SEA1, SEA2 or CMVpp65 (Figure 7), These results indicated that both the proteasome and Sec61 were virtually essential to both MHC Class II- and Class I-directed presentation of exogenous Ag by PBMC [73, 75].

Since previous reports have described an association between proteasome-dependent MHC Class II processing and MHC recycling [76, 77], we examined the impact of the MHC Class II recycling inhibitor, primaquine [77]. Both CD8+ and CD4+T-cell responses were insensitive to primaquine (data not shown). Finally, we also tested the endosomal acidification inhibitor, chloroquine (CQ) [68, 78]. Ag-processing was CQ insensitive (Figure 7), consistent with previous reports that professional APC avoid rapid acidification of exogenous Ags to better preserve intermediate degraded epitopes for Ag-presentation [79]. CQ insensitivity was observed both for GM and for rhGM+R848+LPS conditioning, and occurred whether the restimulatory Ag was SEA1, SEA2 or CMVpp65 (Figure 7),

Identical impacts of the inhibitors were observed whether tested for IFNγ production in restimulatory ICC assays in the presence of the Golgi inhibitor monensin, or assayed by ELISA of supernatants for IFNγ secretion in the absence of monensin (data not shown).

### IL-7 and non-IL-7 driven, GM+R848+LPS conditioned PBMC display indistinguishable kinetics during the initial week of culture

**Figure 8:**
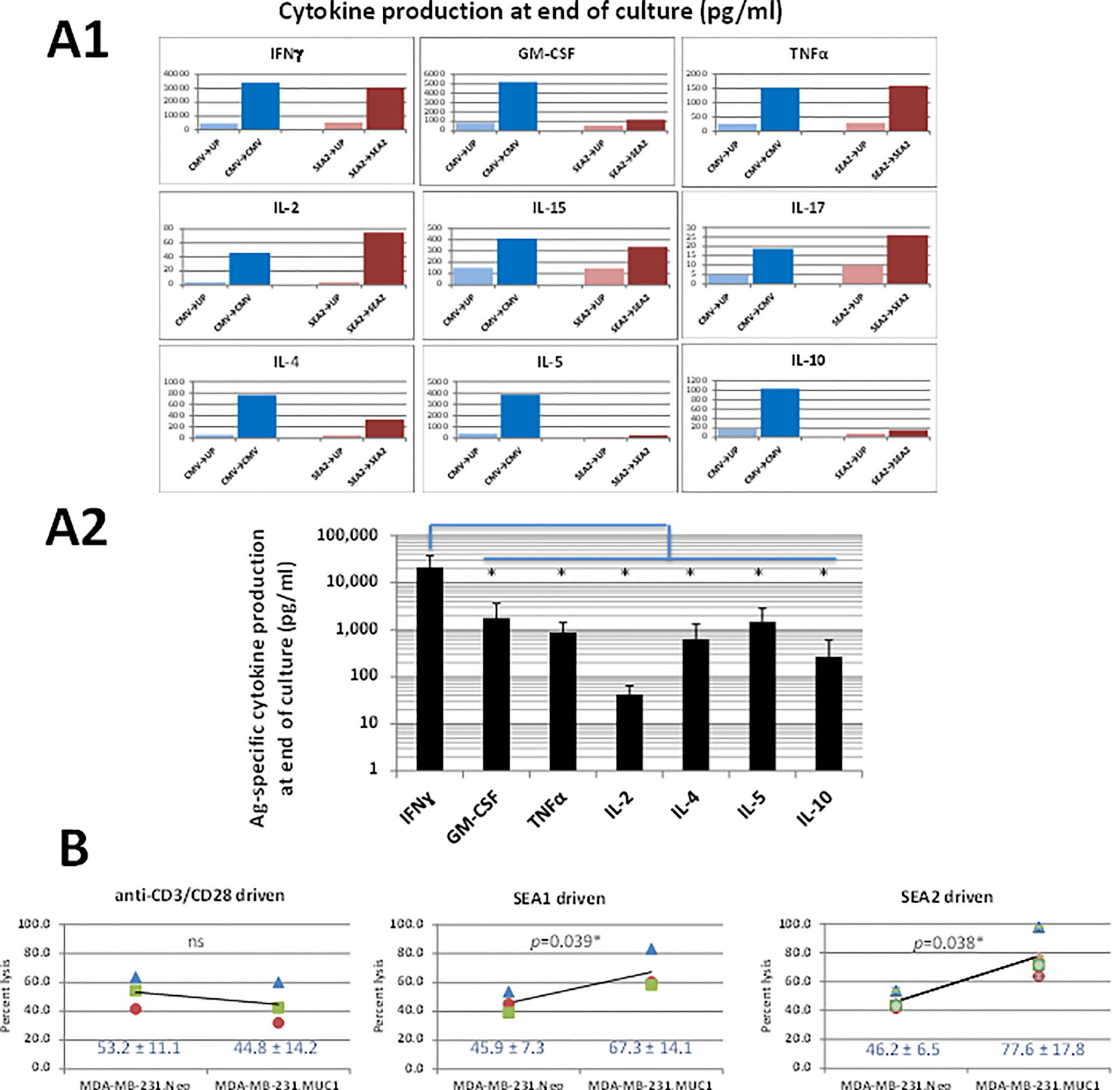
Kinetics of Ag-specific T-cell survival and proliferation attributable to rhIL-7 **A**. PBMC were CFSE labelled on d0 of culture, then conditioned with GM+R848+LPS and either unpulsed or pulsed with CAN, then transitioned to recombinant human rhIL-7+rhIL-2 versus rhIL-2 alone beginning on d2 of culture. In this analysis, representative of four biological replicates, T-cell proliferation over time is indicated by the right-to-left-shift as cell division dilutes intracellular CFSE. **B**. Left panel displays % viable T-cells over time. No significant differences were observed prior to d12, whereas at d12 and d16 viability remained significantly higher for T-cells exposed to CAN, rhIL-7 and rhIL-2 compared to CAN and rhIL-2 without rhIL-7. Right panel demonstrates that the superior viability of the CAN, rhIL-7 and rhIL-2 group from d12 forward corresponded to sustained numeric expansion significantly greater than when rhIL-7 was omitted. Statistical calculations in both panels of **B**. show averaged results of four biological replicates. **C**. Fate of individual PBMC constituents during optimized Ag-driven cultures. PBMC were labelled on d0 with Cell Trace Violet (CTV), conditioned with GM+R848+LPS, pulsed with CAN, then transitioned to rhIL-7 plus rhIL-2. Graph shows in log scale the absolute proportions and numbers of PBMC subpopulations over time, distinguishing CD33+ myeloid cells, never proliferated T-cells (undiluted CTV), and already proliferating T-cells (i.e., CTV diluted due to previous cell division). Data are representative of two biological replicates. **D**. T-cell cultures were labelled with CTV at d9 to facilitate monitoring of continuing proliferation after that time point in conjunction with live-dead staining. Panel representative of two biological replicates displays d16 of culture, with dot plots showing unproliferated dead cells (RUQ), unproliferated live cells (RLQ), proliferated dead cells (LUQ) and proliferated live cells (LLQ). At the d16 timepoint the continuing inclusion of rhIL-7 has licensed sustained high viability **B**.,**D**. and proliferation **A**.,**D**. resulting in marked net numeric T-cell expansion **B**.,**C**. as well as further enrichment of CAN-specific CD4+ and CD8+ T-cells (Figure 2).

To assess the mechanism(s) behind rhIL-7's co-licensure of GM+R848+LPS conditioning, we tracked gross culture expansion and also labeled PBMC with CFSE or its congener Cell Trace Violet (CTV) on d0 to monitor the proliferation of individual T-cells. Unexpectedly, exposure of Ag-pulsed, GM+R848+LPS conditioned PBMC T-cells either to IL-7+IL-2 or to IL-2 alone resulted in indistinguishably high frequencies of proliferation (i.e., CFSE dilution) between days 5 and 8 of initial culture (Figure 8A). In fact, the intense proliferation detectable after d5 already left most dividing T-cells with only background levels of CFSE by d8 (Figure 8A). In contrast, GM+R848+LPS conditioned PBMC subsequently maintained in IL-7+IL-2 but never Ag-pulsed displayed much more sluggish T-cell CFSE dilution out to d8 (Figure 8A). Nonetheless, percent cell viability and overall yields were not significantly different among these three tested conditions during the first 8-9 days of culture (Figure 8B left and right panels).

We sought to reconcile the intense CFSE or CTV dilution of Ag-pulsed PBMC T-cells already observed by d8-9 of culture with the lack of gross numerical expansion up to that time point. By correlating the frequencies of T-cell and myeloid lineage markers with CFSE or CTV tracking and gross numeric expansion, we were able to extrapolate absolute numbers of resting T-cells (undiluted CFSE or CTV) from already proliferating T-cells (diluted CFSE or CTV) as well as CD33+ myeloid cells out to d16 of culture (Figure 8C). Such analyses of GM+R848+LPS conditioned, Ag-pulsed, IL-7+IL-2 treated PBMC revealed that while the CD33+ myeloid fraction did not detectably proliferate (data not shown), it displayed relative enrichment between d0 and d2 of culture due to T-cell dropout. After that, the myeloid fraction itself commenced to drop out, such that the CD33+ fraction was reduced in absolute numbers by approximately half between d2 and 7 in culture, and decimated by d16 (Figure 8C). In contrast, after the first two days of culture, the absolute number of resting T-cells (undiluted CTV) remained virtually unchanged out to d16, indicating that an undetectably small subset of resting T-cells made the transition to proliferation (Figure 8C). Furthermore, the proliferating (diluted CTV) T-cell compartment began to detectably expand in absolute number by d4 of culture, becoming the dominant population after d8 of culture (Figure 8C).

### IL-7 and Ag exposure condition PBMC T-cells for late-stage Ag-specific proliferation and survival protection

Between days 9 and 16 of culture, GM+R848+LPS conditioned, Ag-pulsed PBMC cultures exposed to IL-7 or IL-7+IL-2 displayed a burst in absolute numbers that was solely attributable to continued numeric expansion and high maintained viability of the already proliferating T-cell subpopulation (Figure 2A-2C, Figure 8A-8C). In contrast, GM+R848+LPS conditioned, Ag-pulsed PBMC T-cell cultures exposed to IL-2 without IL-7 displayed a significant decline in viability and only marginal gross numeric expansion after d9 of culture (Figure 2, Figure 8A, 8B).

Because CFSE or CTV labeling of PBMC on d0, d2, or d4 of culture did not permit continuing proliferation analysis after d8-9 of culture, due to many T-cells having already proliferated to background CFSE or CTV levels (Figure 8A and data not shown), we also carried groups in culture for which such labelling was delayed until d9, to prospectively distinguish further proliferation of T-cells and their survival (Figure 8D).

When GM+R848+LPS conditioned, Ag-pulsed PBMC T-cell cultures were exposed to IL-2 without IL-7, they displayed profoundly stunted gross expansion, proliferation and viability after d9 of culture (Figure 8B, 8D). With greater than 80% of cells dead by d16, the surviving T-cells, both CD4+ and CD8+, nonetheless continued to display Ag-specificity in ICC assays (10-40%, Figure 2A, 2B), but with scant evidence of proliferation from d9 forward (Figure 8B, 8D). In contrast, GM+R848+LPS conditioned, Ag-pulsed PBMC T-cell cultures exposed to IL-7 in addition to IL-2 displayed 80% or greater viability at d16, with a sustained proliferating compartment after d9 that demonstrated up to 80% Ag-specificity in ICC assays (Figure 2A, 2B and 8A-D). Therefore, exposure to IL-7 rescued the expanded T-cells from a lethal contraction during the second week of culture, and furthermore licensed continued CD4+ and CD8+ T-cell Ag-specific proliferation with excellent viability.

Recent reports have suggested that lymphopenia-induced homeostatic proliferation favors survival and expansion of Ag-stimulated T-cells due to the ability of endogenous IL-7 to rescue TCR-triggered T-cells from a proapoptotic state [80–83]. Although T-cell upregulation of the anti-apoptotic factor Bcl-2 is a frequently reported impact of IL-7 exposure [84, 85], we have observed that either IL-2 or IL-7+IL-2 treatment induced comparable levels of Bcl-2 in T-cells which proved to be equally well sustained, even when T-cells exposed to IL-2 but not IL-7 were massively dying during week 2 of culture (data not shown). Therefore, rhIL-7's protection of GM+R848+LPS conditioned T-cells does not correlate simply to Bcl-2 induction.

Finally, we sought to determine whether the timing of IL-7 addition to GM+R848+LPS conditioned PBMC cultures was critical to the emergence of T-cell Ag-specificity [32, 35]. Interestingly, indistinguishable synergy between IL-7 and GM+R848+LPS conditioning was observed whether IL-7 was standardly introduced to culture on d2 (after GM+R848+LPS conditioning and Ag pulsation), on d0 of culture (concurrent with GM exposure), on d1 of culture (concurrent with Ag pulsation) or delayed until d4 (data not shown). Furthermore, we tested different concentrations of IL-7 and observed that preferential expansion and survival of Ag-driven T cells was IL-7 dose and schedule dependent, working best when dosing was sufficiently high or frequent to render T-cell expression of the IL-7 receptor (CD127) undetectable for the duration of the culture (data not shown and [86, 87]). Taken as a whole, these data suggest that Ag-driven T-cells emerging from their initial proliferative burst are IL-7 responsive, itself a property associated with long-lived memory T-cells [86, 87].

## DISCUSSION

Historically it has proved difficult to raise large numbers of Ag-specific T-cells from peripheral blood for purposes of adoptive immunotherapy. For example, it has been reported that highly specific anti-MART1 CD8+ T-cells can be propagated from melanoma patients’ peripheral blood, potentially useful when tumor nodules are unavailable to establish TIL cultures. Unfortunately, the robust expansion of the peripheral blood T-cells during many weekly cycles of MART1 peptide pulsing was not adapted to bulk culture methods, but rather extrapolated from scaled-back cultures run in 96- or 24-well cluster plates [12]. While there have been many additional reports that peripheral blood T-cells can be sensitized to recognize tumor-associated Ags by repetitive peptide stimulations, the demonstration of Ag-specificity is often within the context of sluggish cultures already evincing features of T-cell exhaustion [13].

Another historical issue is that the processing of exogenous Ag by PBMC cultures typically results in MHC Class II- but not MHC Class I-restricted presentation, unless the Ag is 8-9 amino acids in length and able to bind directly to MHC Class I clefts already on the cell surface. To get around this requirement, individual laboratories have resorted to rupturing endosomes/lysosomes to leak larger Ags into the cytoplasm where they can access proteasome processing. This was first accomplished in the 1990's by having APC ingest Ag-loaded abrasive iron beads or pH-sensitive liposomes, or by performing transient hypoosmotic shock [88, 89]. Such workarounds have constituted an important proof-of-principle but have provided few clues as to the physiologic signals which might naturally give rise to MHC Class I Ag cross-presentation.

Finally, even though therapeutically active TIL from melanoma patients are more reliably expandable in culture than peripheral blood T-cells, it has not proved possible to reliably expand even TIL unless they are propagated in exceedingly high concentrations of IL-2 (6,000 IU/ml) [1, 2], a maneuver which has not proved to be successfully adaptable to peripheral blood T-cells.

Despite these frustrating culture constraints, the use of adoptive T-cell therapy to treat mouse tumors and human melanoma has historically outperformed vaccine strategies [90], at least in part because the imprecise transition from deficient to effective immunity during vaccine maneuvers itself provides a window of opportunity for tumors to escape by reducing their expression of Ag, MHC molecules, and/or beta2-microglobulin (i.e., immunoediting) [38]. In contrast, temporarily removing T-cells from the body to optimize their number and synchronize their activation state prior to adoptive reinfusion can markedly improve their subsequent *in vivo* therapeutic performance. This is clearly illustrated by reports that culture-activated T-cells from pmel mice (transgenic for TcR recognizing the melanoma-associated Ag gp100) can be given in a dose-dependent manner as adoptive therapy with vaccinia-gp100, rIL-2 and sublethal irradiation to cure normal syngeneic B6 mice of sizable established B16 melanoma challenges. In contrast, adoptive transfer of non-culture-activated pmel T-cells is ineffective [91]. Furthermore, pmel mice themselves do not resist B16 challenges [91].

The present culture system demonstrates that unvaccinated donor PBMC pulsed with peptide sequences derived from MUC1, HER2, and CMVpp65 can rapidly and consistently give rise to high frequencies and yields of predominantly T1-type T-cells, both CD4+ and CD8+, that recognize these antigens. Furthermore, MUC1-sensitized T-cells preferentially lysed partially histocompatible MUC1-expressing tumor targets. Our success driving cultures with longer peptides demonstrates that PBMC contain APC which can routinely process and cross-present complex Ags to CD8+ T-cells, in addition to presenting them to CD4+ T-cells. Optimal culture outcomes are observed so long as the necessary licensing factors—rhGM, R848, LPS and rhIL-7—are included along with driving Ag.

We ascertained that upfront conditioning of extracorporealized PBMC with GM+R848+LPS to simulate a life-threatening infection has no apparent detrimental impact on T-cell function while it profoundly enhances APC function. We and others have identified that decisive activation of innate immunity either *in vitro* or *in vivo* requires exposure to two or more closely timed danger signals [25–27]. Activation of robust IL-12 and IL-23 production is a signature event of strongly activated innate immunity and DC1 polarization, which necessitates the activation of myeloid DC or monocytic DC precursors rather than IFNα-secreting plasmacytoid DC [25]. We observed that, for unfractionated human PBMC, the combination of the MyD88-activating TLR8 agonist R848 and the TRIF/MyD88-activating TLR4 agonist LPS was far superior to other danger signal pairings for generating reliable and robust IL-12p70 production, particularly when GM conditioning preceded TLR agonist exposure. Furthermore, the abundant secretion of IL-12p70 was accompanied by equally abundant IL-23 secretion, significant upregulation of B7.1 and CD40 expression, and *de novo* expression of additional co-stimulatory factors (CD70 and 4-1BBL).

Because CD33+ PBMC can readily be differentiated into myeloid DC by a variety of treatments [19–21, 25–27], it is not surprising that, even without exposure to danger signals, they utilize an alternative, endosome-centric ERAD-like pathway to professionally isolate the processing of exogenous Ag from endogenous Ag. It is likely that the classical BFA-sensitive ER-based pathway, though accessible to exogenous Ag transiting the cytosol, prioritizes the detection and sterilization of endogenous pathogens. Furthermore, the observed resistance of exogenous Ag processing to CQ indicates that this non-classical ERAD-pathway avoids precipitous lysosomal acidification which can eradicate pathogens, but can also excessively catabolize peptides until they are too short to bind to MHC clefts. Instead, the processing of exogenous Ag for both MHC Class I and Class II presentation appears to invoke a Sec61-dependent round trip between endosome and cytosol to access proteasomally controlled degradation. Even though this professional routing of exogenous Ag is observed for both GM+R848+LPS conditioning and GM conditioning alone (Figure 7), GM+R848+LPS is far superior for initial launching of Ag-driven T-cell expansion (Figure 1D and 6B), likely due to its distinctive upregulation of costimulatory molecules and DC1-polarized cytokine production (Figure 6A).

Because MUC1 and HER2 are, in composite, hyperexpressed by the majority of human cancers [38, 92], the ability to readily grow out natural T1-type CD4+ and CD8+ T-cells recognizing these Ags, even from the PBMC of non-vaccinated donors, has the potential to make adoptive natural T-cell therapy applicable to most cancer patients, also potentially bypassing the need for vaccine maneuvers which themselves could prematurely result in tumor escape through immunoediting [38]. It is encouraging that autologous adoptive therapy with natural EBV-specific PBMC-derived T-cells has already proved to be a well-tolerated and clinically effective therapy for lymphoma patients whose tumors express EBV product [22]. We have already confirmed that our culture method for targeting MUC1 and HER2 can be successfully scaled up for the purpose of treating patients (Supplemental Figure S5B). Furthermore, preliminary PBMC cultures established from breast cancer patients confirm that our culture system and antigen discovery algorithm can be extended successfully to patients with MUC1- and HER2-expressing malignancies (Supplemental Figure S5A). Finally, the likely physiologic expression of checkpoint receptors such as PD-1 and CTLA-4 on subsets of the culture-expanded T-cells (Supplemental Figure S3) suggests that co-treatment with checkpoint inhibitors may substantially potentiate the T-cells’ continued expansion and survival *in vivo* post-infusion.

## MATERIALS AND METHODS

### Ethics statement

Investigations have been conducted in accordance with the ethical standards of the Declaration of Helsinki, as well as current national and international guidelines, and have been approved by the authors’ institutional review boards. Data reported in this article involving the blood cells of both healthy donors and cancer patients were performed under Mayo IRB #09-000263 and IRB #15-007-402, in each case with informed consent obtained.

### Reagents used in culture

#### Media

Many culture media were comparatively screened for their capacity to support propagation of natural Ag-specific T-cells from human PBMC, including RPMI 1640 (Thermo-Fisher, Waltham MA, #11875119), CTS AIM-V (Thermo-Fisher #0870112DK), CTS OpTmizer (Thermo-Fisher #A1048501), *Ex Vivo*10 (Lonza, Walkersville MD, #04-380Q), and Gibco macrophage SFM (Thermo-Fisher #12065074), variously supplemented with human AB serum (HuAB) already heat-deactivated at purchase (Fisher-Scientific/Gemini #100-512, Lot#H15M03A). Optimal culture performance was attained employing AIM-V already formulated to contain glutamine, penicillin and streptomycin, supplemented only with Gibco Amphotericin B at an optimized final concentration of 0.125 μg/ml (Thermo-Fisher #15290018). Serum-free T-cell propagation was not sustainable, but supplementation of AIM-V with 0.5% HuAB during Step 1 (d0-2 of culture) and 2.0% HuAB during Step 2 (d2 and thereafter) proved optimal for robust expansion of both CD4+ and CD8+, natural Ag-specific T-cells. Culture incubators were maintained at a CO_2_ tension of 5%.

#### Step 1 factors

As shown in Figure 1B, agents added standardly during Step 1 (d0-2 of culture) included recombinant human (rh) GM-CSF on d0 of culture (sargramostim, Sanofi-Aventis, Bridgewater NJ, optimized final concentration 40 ng/ml); resiquimod on d1 of culture (R848 VacciGrade, Invivogen, San Diego CA, optimized final concentration 3 μg/ml); and LPS on d1 of culture (E. coli 026:B6, Sigma-Aldrich, St Louis MO, #2654, optimized final concentration 5 ng/ml). Other agents tested in Step 1 but absent from the optimized formulation included rhIL-4 (Peprotech, Rocky Hill NJ, #200-04. 20 ng/ml); rhIL-12 (Peprotech #200-12, 10 ng/ml); rhIFNγ (Actimmune, Horizon Pharma, Deerfield IL, 2000 IU/ml); and polyI:C (Sigma-Aldrich #P1530, 50 μg/ml). Ags added on d1 of culture (prior to R848 and LPS) included MUC1-, HER2/neu- and CMVpp65-derived synthetic peptides added at an optimized final concentration of 50 μg/ml when pulsed singly, or 10 μg/ml each when multiple peptides were pulsed as a cocktail (see Peptide synthesis below); clinical grade Candida albicans extract (CAN, Hollister Stier, Spokane WA, #5053, at a 10% v:v optimized final concentration); and recombinant HER2/neu intracellular domain protein (HER2-ICD, SignalChem, Richmond,BC #E27-11G at an optimized final concentration of 50 μg/ml).

#### Step 2 factors

Beginning with culture Step 2 (d2 of overall culture), rhIL-7 (Miltenyi Biotec premium grade, Auburn CA, #130-095-364) was added at an optimized final concentration of 50 ng/ml to fresh medium added at culture splits. Other agents tested in Step 2 but absent from the optimized formulation included rhIL-2 (aldesleukin, Prometheus Labs, San Diego, CA, final concentration 24 IU/ml); rhIL-15 (Peprotech #200-15, final concentration 5 ng/ml) and rhIL-21 (Peprotech #200-21, final concentration 3 ng/ml).

#### Ag processing inhibitors

When thawed cryopreserved PBMC were pulsed with Ag for use as restimulators of culture-expanded autologous T-cells, they were also electively exposed to standard doses of the following Ag-processing inhibitors: brefeldin A (Sigma-Aldrich #B5936-200UL, optimized final concentration 10 μg/ml); MG132 (Calbiochem/EMD Millipore, Billerica MA, #474791, optimized final concentration 10 μM); lactacystin (Santa Cruz Biotech, Santa Cruz CA, #SC-3575, optimized final concentration 10 μM); exotoxin A (Sigma-Aldrich #P0184, optimized final concentration 100 ng/ml); and chloroquine (Santa Cruz Biotech #SC-205629, optimized final concentration 100 μM). Inhibitors were thoroughly washed out before the restimulating PBMC were combined with already culture-expanded T-cells.

### Peptide synthesis

The following long peptides were synthesized for immunotargeting in the Mayo Peptide Synthesis Laboratory or at Genscript (Piscataway NJ), based on analyses indicating a wealth of embedded peptides with predicted high affinity for multiple HLA-DR haplotypes as well as for HLA-A2.1 (see Results and Figure 3A, 3B and 4A):

CMVpp65-derived 28mer (SQEPMSIYVYALPLKMLNIPSINVHHYP)

MUC1-SEA domain-derived 32mer (SEA1) (SPQLSTGVSFFFLSFHISNLQFNSSLEDPSTD-amide}

MUC1-SEA domain-derived 39mer (SEA2) (STDYYQELQRDISEMFLQIYKQGGFLGLSNIKFRPGSVV-amide)

HER2 p146-174 29mer (TEILKGGVLIQRNPQLCYQDTILWKDIFH)

HER2 p848-865 21mer (DLAARNVLVKSPNHVKITDFG)

HER2 p675-703 29mer (IKRRQQKIRKYTMRRLLQETELVEPLTPS)

HER2 p776-797 22mer (GVGSPYVSRLLGICLTSTVQLV)

MUC1-VNTR 24mer was also synthesized as a control for its contrastingly low predicted affinities for HLA-DR and A2.1 (Figure 3A) (AHGVTSAPDTRPAPGSTAPPAHGV-amide).

All peptides were produced to > 98% homogeneity as confirmed by mass spectrometry.

### Collection and preservation of PBMC

A total of 7 healthy HLA-A2.1+ eligible volunteer donors consented to undergo repetitive leukapheresis collections at intervals deemed safe and appropriate by the American Association of Blood Banks. Collections were performed using antecubital access and return, employing the COBE Spectra apheresis system with settings to minimize retention of RBC and neutrophils in the collection. Occasional collections notable for visible RBC retention were subjected to Ficoll-Hypaque density separations (Ficoll-Paque Plus, Thermo-Fisher #17-1440-02) for 20 minutes at 450 x g (2,000 rpm) to isolate the RBC- and neutrophil-poor PBMC interface. PBMC collections were observed to perform well in culture whether or not they required the Ficoll-Hypaque step (data not shown). All collections were then repetitively washed more slowly at 115 x g (1,000 rpm) in PBS to eliminate platelets from the pelleting PBMC, endpointed by an absence of turbidity in the supernatant. Cells were then resuspended in ice cold HuAB, to which an equal volume of 20% DMSO/80% HuAB was added dropwise for a final concentration of 10% DMSO (Sigma-Aldrich #D2438). Aliquots in cryovials were placed overnight in a freezer at -80^o^ C prior to transfer into liquid nitrogen storage. The performance of all PBMC detailed in this report reflects PBMC which were subjected to initial cryopreservation on the collection day, followed days to months later by thaw and culture. Up to 12 billion PBMC were safely collected during each leukapheresis procedure.

PBMC were also collected from cancer patients consenting to a single 100 ml peripheral venipuncture, always followed by a Ficoll-Hypaque density separation for RBC and neutrophil depletion.

### Optimized PBMC culture and restimulation assays for propagation of naturally Ag-specific T-cells

For limited scale T-cell expansions as in Figure 1B, thawed PBMC on d0 of culture (i.e., Step 1) were resuspended in AIM-V with 0.5% HuAB and 40 ng/ml rhGM-CSF (rhGM) at 6 million PBMC/ml, and plated at 1 ml per well in 24-well cluster plates (Costar Corning/Sigma-Aldrich #3524). The next day (d1 of culture) wells were Ag-pulsed, and exposed 4h later to R848, then 30m later to LPS. The next day (d2 of culture, beginning of Step 2) cells were harvested, with cell detachment facilitated by washing with Ca/Mg-free PBS. Cells were centrifuged, washed again in PBS, resuspended in AIM-V with 2% HuAB and 50 ng/ml rhIL-7 to 12 times the initial volume, and plated at 2ml/well in fresh 24 well cluster plates. Further intermittent splits including fresh rhIL-7 were performed upon yellowing of the media, with final harvest performed on d16 to d19. 2d prior to final harvest, cryopreserved autologous PBMC were freshly thawed and subjected to a 2-day “Step 1” culture including Ag pulsing to provide restimulatory PBMC for intracellular cytokine (ICC) assays, Luminex assays, and/or further Ag-driven culture expansion, typically at a 2:1 ratio of harvested T-cells to restimulatory PBMC. The restimulatory PBMC did not require exposure to GM, R848 or LPS to present pulsed-Ag efficiently to the culture-expanded T-cells.

Bulk expansions were also performed using Wilson-Wolf G-Rex 100M (1-liter) vessels (Brighton, MN) instead of 24-well cluster plates.

### ICC and luminex assays

Culture-expanded T-cells were co-cultured with freshly thawed autologous restimulatory PBMC, pulsed either with the Ag which drove the expanded T-cells, irrelevant Ag, or no Ag (unpulsed). Restimulation was performed in 24 well cluster plates with 2 million T-cells and 1 million restimulatory PBMC per well for 18-24h. For ICC, monensin (GolgiStop, BD Biosciences, San Diego CA, #554724) was added per manufacturer guidelines after 4-6h to block export of endogenously produced cytokines, and subsequent co-staining for surface CD4 and CD8 was performed in addition to intracellular cytokine staining to delineate the T-cell subsets’ respective contribution to cytokine production. Luminex-assayed production of cytokines on a multiplex platform (Thermofisher) was performed on supernatants identical to ICC restimulatory cultures except for the exclusion of monensin, following the manufacturer's guidelines.

### FACS analyses

When proliferation in culture was analyzed (e.g. Figure 8), cells were preloaded with cytoplasmic tags which diluted linearly with cell division, using CellTrace CFSE or Violet Cell Proliferation Kits (Invitrogen/Thermo-Fisher, #C34554 and #C34557). Multicolor analyses were performed on a five laser Fortessa (BD Biosciences). Cells were buffered during staining with Ca/Mg-free PBS (Gibco/Thermo-Fisher #10010) containing 1% heat-deactivated fetal bovine serum (FBS, Sigma-Aldrich #F2442) and 0.02% sodium azide (Sigma-Aldrich #S-8032). Fc receptor blockade was performed by preadding 50 μg of unconjugated human IgG (Sigma-Aldrich #I4506) to each sample.

Fluorescently-conjugated mAb targeting cell surface proteins were then added, also including Live/Dead UV Blue Stain to delineate viability (Life Technologies/Thermo-Fisher #L23105). If intracellular proteins were also to be co-analyzed (IFNγ and/or other cytokines, Foxp3, Helios, or CTLA-4), staining for cell surface proteins was followed by fixation and permeabilization per manufacturer guidelines (eBioscience, San Diego CA, #00-5123-43, #00-5223-56 and #00-8333-56; BD Biosciences #51-2090KZ, #51-2091KZ), followed by staining for the intracellular proteins. mAb employed included anti-CD3 APC efluor 780 (eBioscience #47-0036-42); anti-CD4 BV 510 (BD Horizon/BD Biosciences #582970); anti-CD8 evolve 655 (eBioscience #86-0088-42); anti-CD33 APC (eBioscience #17-0338-42); anti-CD56 efluor 710 (eBioscience #46-056-42); anti-CD56 FITC (MEM-188) (eBioscience #11-0569-42) anti-HLA-DR PE (eBioscience #12-9956-42); anti-CD11c eFluor 450 (eBioscience #48-0116-42); anti-IL-12p70 FITC (BD Pharmingen/BD Bioscience #554574); anti-CD80 (B7.1) BV650, BioLegend, San Diego CA, #305227); anti-4-1BB FITC (eBioscience #11-1379-42); anti-4-1BBL PE (BD Pharmingen #559446); anti-CD70 PE (BD Pharmingen #555835); anti-CD40 eFluor 450 (eBioscience # 48-0409-42); anti-IFNγ efluor 450 (eBioscience #48-7319-42); anti-IL-2 FITC (eBioscience #11-7029-42); anti-IL-17A APC-eFluor 780 (eBioscience #47-7179-42); anti-CD25 BB515 (BD Horizon #564467); anti-CD28 PerCP-Cy5.5 (eBioscience #45-0289-42); anti-CTLA-4 BV421 (BD Horizon #562743); anti-PD-1 BV 785 (BioLegend #329930); anti-Foxp3 PE-Dazzle 594 (BioLegend #320126); anti-Helios PerCP-Cy5.5 (BioLegend #137230); anti-LAP APC (eBioscience #17-9829-42); anti-GARP BV421 (BD Horizon #563956). Appropriate isotype controls were also acquired for each evaluated specific mAb.

### ELISA assays

Culture supernatants were assayed for IL-12p70 and/or IL-23 content employing ELISA reagents from eBioscience (San Diego CA), following manufacturer guidelines.

### Lytic assays

PBMC from multiple healthy HLA-A2.1+ donors were either driven polyclonally in culture with immobilized anti-CD3 plus soluble anti-CD28, 1 μg/ml and 0.5 μg/ml respectively (BD Pharmingen #555329 and #555725), or driven by SEA1 or SEA2 long peptides synthesized from the SEA domain of MUC1 with GM+R848+LPS+IL-7 conditioning. At the end of culture, SEA-driven T-cells selectively recognized the driving SEA peptides when reexposed to peptide-pulsed autologous stimulatory PBMC (e.g., Figure 3) whereas polyclonally-driven T-cells did not (data not shown). To determine whether MUC1-expressing malignant cells could also be targeted, we employed the HLA-A2.1+ human breast cancer line MDA-MB-231, purchased from the American Type Culture Collection, stably transduced with retroviral vector pLNCX.1 expressing MUC1 (MDA-MB-231.MUC1) or Neo control (MDA-MB-231.Neo) [93]. Analyses by IDEXX (Columbia MO) confirmed the human origin of the cell lines, absence of mycoplasma, and that the MUC1+ and Neo control cell lines originated from ATCC HTB-26. Staining with anti-MUC1 mAb (FITC-anti-CD227, clone HMPV, BD Biosciences) confirmed cell surface staining for MDA-MB-231.MUC1 but not MDA-MB-231.Neo. The malignant cell lines were incubated overnight with 0.5 ng/ml recombinant human IFNγ to enhance their MHC expression (BD Bioscience #554617) prior to labelling with Cr^51^ (200 μCi, Perkin Elmer, Bridgeport CT, #NEZ03005MC) for 3 hrs. Each of the T-cell groups was then cultured for 8h with each of the Cr^51^-labelled malignant lines at a 100:1 ratio, then supernatants assayed on a Top Count NXT counter (Perkin/Elmer). % Lysis was calculated as ((Experimental Lysis - Spontaneous Cr^51^ release)/(Complete Lysis in Triton X-100 - Spontaneous Cr^51^ release)) x 100.

### Statistical analysis

Biological replicability was confirmed for all experiments as designated in the figure legends. Analyses by Student *t*-test were always two-tailed, and unless identified as paired data were performed unpaired. Comparisons determined to be *p* < 0.05 but > 0.01 were labelled *, *p* ≤ 0.01 but > 0.001**, *p* ≤ 0.001 but > 0.0001***. and *p* ≤ 0.0001****. ns = not significant. In Figure 2 a multiple linear regression analysis was performed as detailed in Supplemental Table S1 and Figure 2F.

## SUPPLEMENTARY MATERIALS FIGURES AND TABLES



## Abbreviations

Ag: antigen(s)
AIT: adoptive immunotherapy
APC: antigen-presenting cell(s)
BFA: brefeldin A
CFSE: carboxyfluorescein succinimidyl ester
CQ: chloroquine
CTV: Cell Trace Violet
CMV: cytomegalovirus
ER: endoplasmic reticulum
ERAD: ER-associated degradation (pathway)
ExoA: exotoxin A
GM: GM-CSF
HER2: HER2/neu (ERBB2)
HuAB: human AB serum
ICC: intracellular cytokine (assay)
ICD: intracellular domain of HER2/neu
IL-12p70: assembled IL-12 dimer
LPS: lipopolysaccharide
LT: lactacystin
LUQ/LLQ/RUQ/RLQ: left/right upper/lower quandrant (dot plots)
MFI: mean fluorescent index
PBMC: peripheral blood mononuclear cells
R848: resiquimod
rh: recombinant human
SEA: Sperm protein, Enterokinase and Agrin domain of MUC1
VNTR: Variable Number of Tandem Repeats domain of MUC1
TAP: Transporter-Associated with Ag Processing
TIL: tumor-infiltrating lymphocytes
TLR: toll-like receptor (agonist)
Treg: regulatory T-cell
