# Supplementary material for: Surrogate in vitro activation of innate immunity synergizes with interleukin-7 to unleash rapid antigen-driven outgrowth of CD4+ and CD8+ human peripheral blood T-cells naturally recognizing MUC1, HER2/neu and other tumor-associated antigens
Source: Oncotarget. 2016 Dec 11;8(7):10785–808. doi: 10.18632/oncotarget.13911 (PMC5355224; doi:10.18632/oncotarget.13911)
Supplement: Supplementary file 1 [file oncotarget-08-10785-s001.pdf]

Surrogate *in vitro* activation of innate immunity synergizes with interleukin-7 to unleash rapid antigen-driven outgrowth of CD4+ and CD8+ human peripheral blood T-cells naturally recognizing MUC1, HER2/neu and other tumor-associated antigens

Supplementary Material

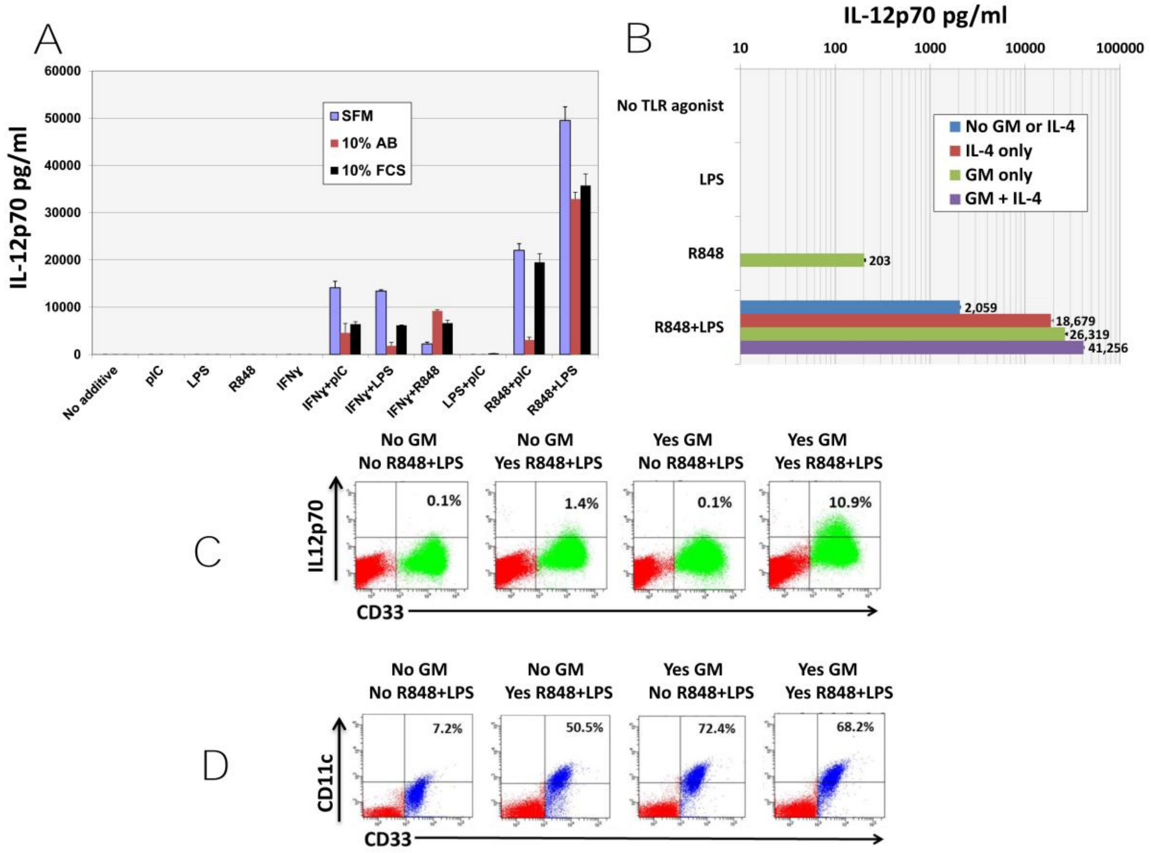

Supplemental Fig S1: Functional activation of CD33+ (myeloid) PBMC during the first 2 days of culture: A. IL-12p70 production by CD33+ PBMC is strongly impacted by culture media. Cultures as in **Fig 1A**, comparing impacts of media upon danger signal-induced IL-12p70 production. Complete RPMI 1640 supplemented with 10% heat-deactivated human AB serum was compared to RPMI 1640 10% FCS, and to Gibco macrophage SFM, all groups receiving GM-CSF (GM) and IL-4 on d0 and various danger signals on d1 for a d2 harvest. Representative of two biological replicates. **B.** Culture as in **Fig 1A**, comparing impacts of 40 ng/ml GM and/or 20ng/ml IL-4 upon danger signal-induced IL-12p70 production in RPMI 1640 with 10% human AB serum. Representative of two biological replicates. Note that IL-12p70 production is shown in log scale. **C.** d2 PBMC stained for surface CD33 and intracellular IL-12p70 demonstrating that IL-12p70 production was confined to the CD33+ myeloid fraction (green subpopulation). Right upper quadrants (RUQ) show % of CD33+ cells that are also IL-12p70+, with background isotype control staining subtracted. **D.** d2 PBMC stained for surface CD33 and surface CD11c, demonstrating that treatment with GM and/or TLR agonists resulted in generalized myeloid cell expression of CD11c (blue subpopulation). RUQ shows % of CD33+ cells that are also CD11c+, with background isotype control staining subtracted. Representative of three biological replicates.

Supplemental Table S1: Calculations for regression analyses in Figure 2

- CD4 Linear Regression
  - R^2 = 0.84244, Adjusted R^2 = 0.77491
  - ANOVA: Test of Strength of Multiple Linear Regression, If valid model, then p<0.02
    - p=0.00613, so valid model
  - Which Dependent Variable Is Significantly Associated With Response
    - IL2, p = 0.37771
    - IL7, p = 0.00185
    - IL15, p = 0.35285
    - IL21, p = 0.56173

- CD8 Linear Regression
  - R^2 = 0.80289, Adjusted R^2 = 0.71841
  - ANOVA: Test of Strength of Multiple Linear Regression, If valid model, then p<0.02
    - p=0.01295, so valid model
  - Which Dependent Variable Is Significantly Associated With Response
    - IL2, p = 0.58893
    - IL7, p = 0.00572
    - IL15, p = 0.44131
    - IL21, p = 0.79968

- Fold Expansion Linear Regression
  - R^2 = 0.9034, Adjusted R^2 = 0.862
  - ANOVA: Test of Strength of Multiple Linear Regression, If valid model, then p<0.02
    - p=0.00117, so valid model
  - Which Dependent Variable Is Significantly Associated With Response
    - IL2, p = 0.53234
    - IL7, p = 0.00406
    - IL15 , p = 0.35856
    - IL21, p = 0.31246

Supplemental Table S2: 29 HLA-DR haplotypes encompass nearly 90% of individuals. Peptide regions with clusters of embedded 15-17mers displaying high affinity for these haplotypes are designated as promisingly promiscuous "hot spots" for MHC Class II binding.

Supplemental Table 2: Identification of HLA-DR Alleles Across Races and Ethnicities

DRB1 Alleles	African American Frequency	Caucasian Frequency	Chinese Frequency	Hispanic Frequency	Indian Frequency	Japanese Frequency	Korean Frequency
0101	2.65%	8.60%	0.93%	4.33%	3.21%	5.84%	5.78%
0102	3.92%	1.38%	0.07%	3.32%	0.13%	0.06%	0.02%
0301	6.99%	12.16%	6.81%	6.95%	7.46%	0.68%	2.20%
0302	6.31%	0.03%	0.00%	0.50%	0.01%	0.01%	0.00%
0401	2.02%	8.78%	0.51%	1.81%	0.90%	1.15%	0.78%
0403	0.17%	0.79%	2.31%	1.84%	5.27%	2.43%	2.57%
0404	0.82%	3.88%	0.88%	5.76%	2.01%	0.32%	1.39%
0405	1.53%	0.67%	6.12%	2.22%	0.75%	14.72%	8.94%
0407	0.39%	1.12%	0.08%	7.47%	0.13%	0.64%	0.44%
0701	10.11%	13.42%	5.31%	9.61%	16.95%	0.94%	7.15%
0802	0.09%	0.08%	0.55%	9.64%	0.51%	4.34%	2.50%
0803	0.04%	0.24%	6.80%	0.27%	0.71%	7.44%	7.62%
0804	5.42%	0.20%	0.01%	0.68%	0.08%	0.02%	0.00%
0901	2.97%	1.03%	15.54%	0.82%	0.94%	13.87%	9.67%
1001	1.92%	0.85%	1.34%	1.30%	6.28%	0.40%	1.70%
1101	8.54%	5.56%	6.26%	3.55%	5.98%	2.58%	4.73%
1104	0.58%	2.95%	0.25%	3.25%	1.97%	0.12%	0.07%
1201	3.82%	1.64%	3.42%	0.91%	0.60%	3.75%	4.83%
1202	0.29%	0.02%	11.50%	0.15%	2.99%	1.71%	3.45%
1301	5.42%	5.63%	0.78%	3.72%	6.73%	0.76%	1.73%
1302	7.30%	4.88%	2.42%	3.50%	3.37%	5.75%	8.62%
1303	3.26%	1.09%	0.02%	1.06%	0.13%	0.04%	0.01%
1401	1.86%	2.61%	3.33%	1.84%	1.13%	3.01%	2.68%
1404	0.05%	0.07%	0.51%	0.03%	7.13%	0.02%	0.06%
1406	0.01%	0.02%	0.02%	4.27%	0.05%	1.42%	0.68%
1501	2.82%	13.46%	10.12%	6.43%	9.02%	8.67%	7.94%
1502	0.23%	0.72%	2.66%	1.17%	10.73%	9.67%	3.18%
1503	11.66%	0.05%	0.00%	0.58%	0.02%	0.01%	0.00%
1602	1.38%	0.15%	4.35%	2.47%	0.67%	0.67%	0.99%
Total Coverage	92.6%	92.1%	92.9%	89.4%	95.8%	91.0%	89.7%

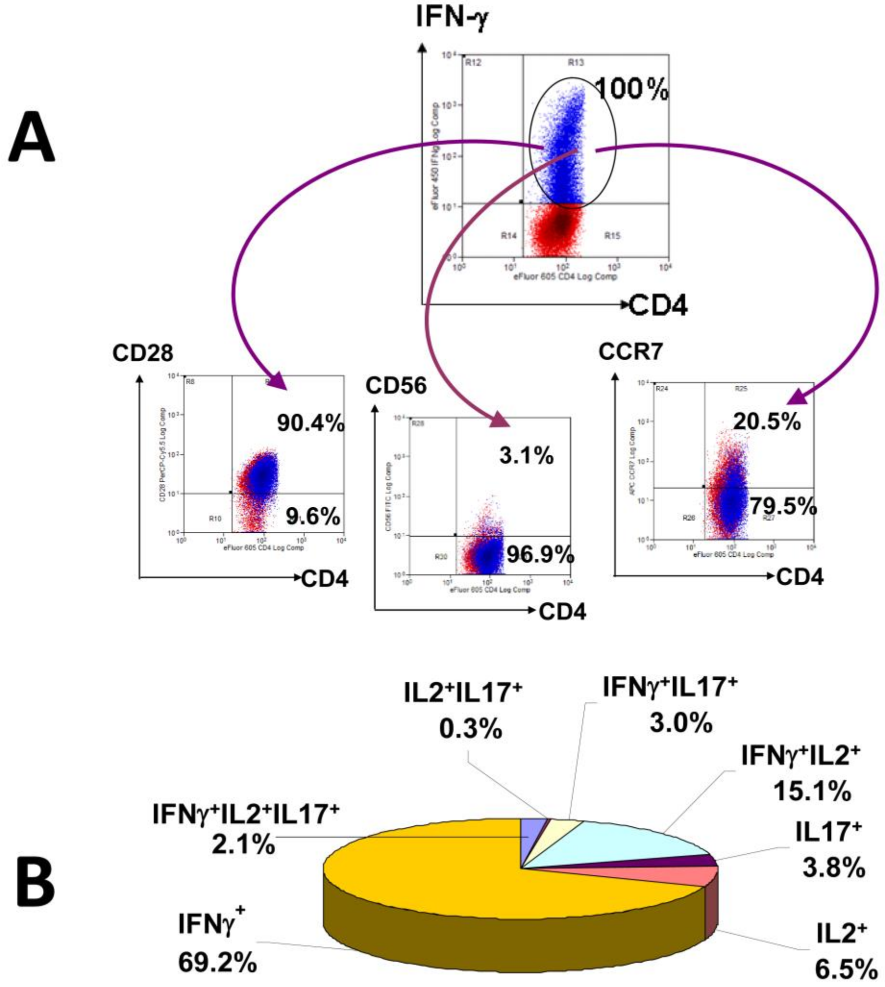

Supplemental Fig S2: Phenotype and functional analyses of culture-expanded PBMC-derived T-cells. CAN-driven, GM+R848+LPS conditioned PBMC-derived T-cell cultures were restimulated with CAN-pulsed PBMC in an ICC assay on d16. Cultures were run as described in **Fig 1B-D**. **A.** Co-expression of functional markers. CD4+ T-cells staining positively for intracellular IFNγ upon CAN restimulation (blue subpopulation) were co-analyzed for CD28, CD56, and CCR7. It was observed that >90% of IFNγ+ cells costained for CD28 and <5% for CD56. Subsets of both CCR7+ and CCR7- IFNγ+ cells were observed, consistent with a mix of central memory and memory effector T-cells. **B.** CD4+ T-cells reexposed to CAN were simultaneously stained intracellularly for IFNγ, IL-2 and IL-17 using distinctive fluorochrome conjugates. Percentages of cells staining positivity for at least one of these cytokines were enumerated. This is representative of 3 experiments examining simultaneous T-cell production of multiple cytokines.

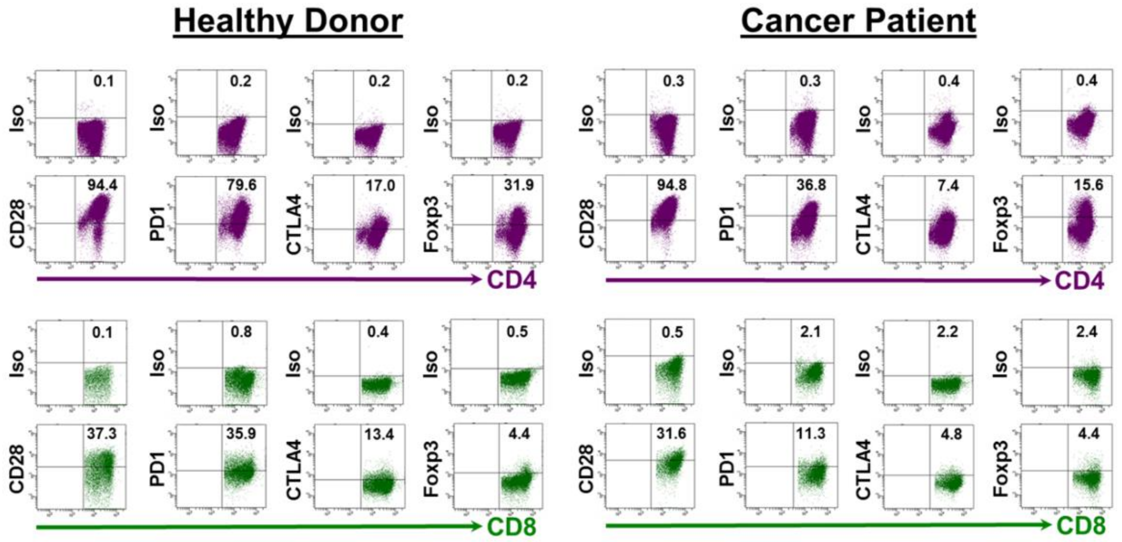

Supplemental Fig S3: CD28, PD1, CTLA4 and Foxp3 expression on T-cells at end of culture. Multicolored FACS analysis of CMVpp65-driven, PBMC-derived T-cell cultures run from a healthy donor and a patient with metastatic breast cancer (cultures performed as in **Fig 1B** “GM+R848+LPS” group), showing T-cell co-expression of CD28, PD1, CTLA4 and Foxp3 (see Methods for staining details). Analyses are gated to show CD4+ or CD8+ cells. Numbers shown in RUQs indicate the % of CD4+ or CD8+ T-cells co-expressing the molecules of interest. Representative of 3 biological replicates.

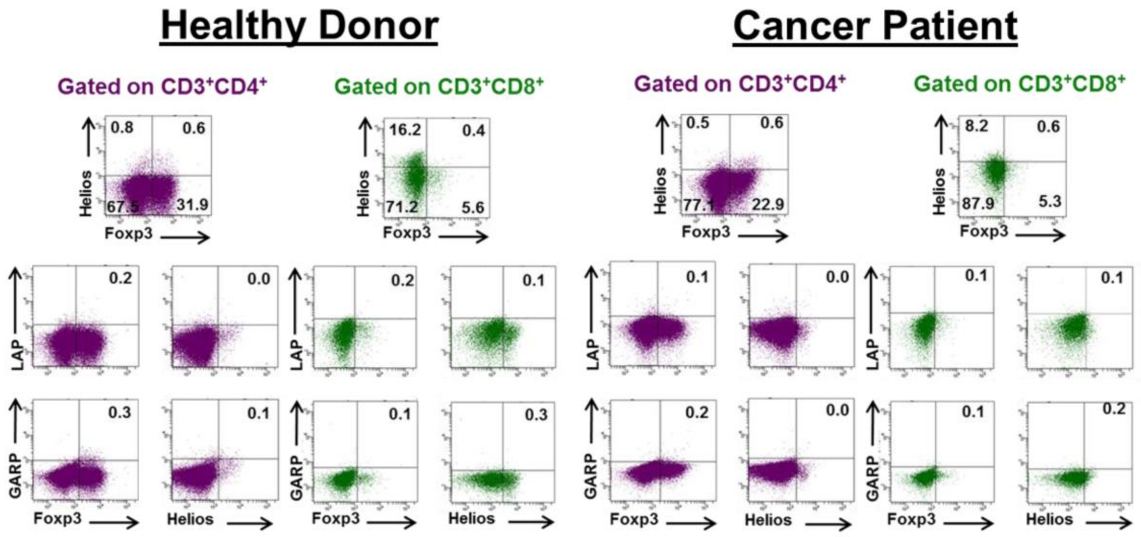

Supplemental Fig S4: Foxp3, Helios, LAP and GARP co-expression on T-cells at end of culture. Multicolored FACS analysis of CMVpp65-driven, PBMC-derived T-cell cultures run from a healthy donor and a patient with metastatic breast cancer (cultures run as in **Fig 1B**, see Methods for staining details). Analyses are gated to show CD4+ or CD8+ cells, with numbers in each right upper quadrant (RUQ) indicating the % of total CD4+ or CD8+ T-cells within the quadrant co-expressing the molecule(s) of interest. Representative of 3 biological replicates.

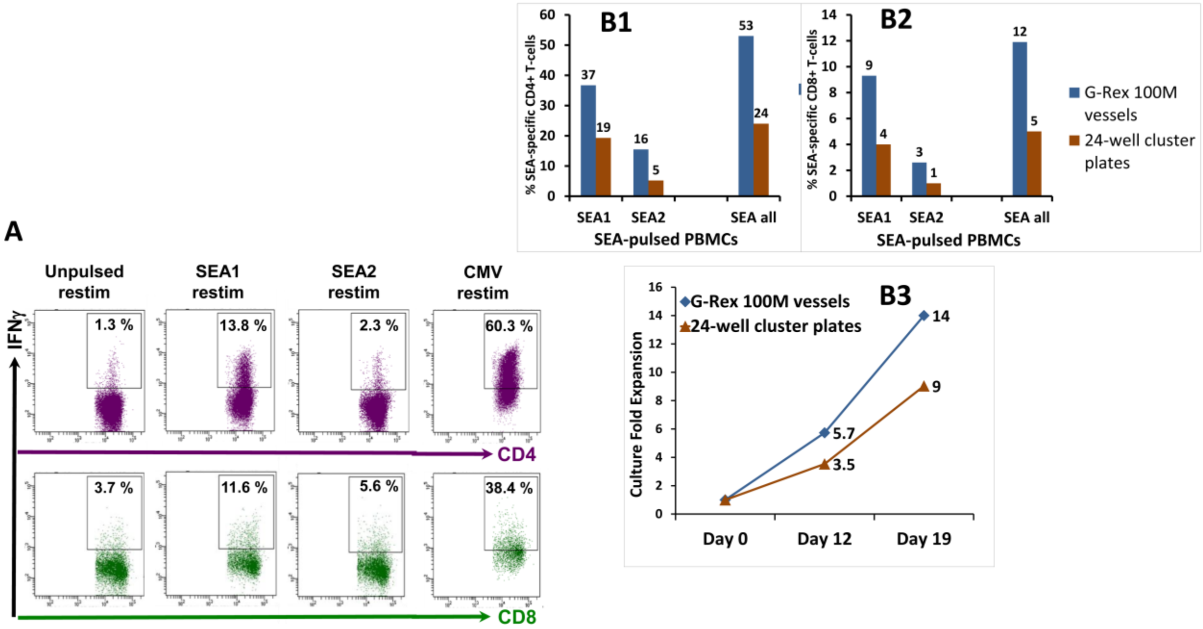

Supplemental Fig S5: T-cells cultured from unfractionated PBMC can be effectively driven by cocktails containing multiple antigenic peptides, and can be effectively expanded in scaled-up culture vessels. A. PBMC were obtained by venipuncture from a patient with advanced breast cancer. With cultures performed as in **Fig 1B** "GM+R848+LPS"" group, PBMC were exposed on culture d1 to a cocktail of SEA1, SEA2 and CMV peptides. Prior experiments demonstrated that while pulsing individual peptides at 50 µg/ml was most effective for driving T-cell sensitization, combining the peptides in a cocktail was most effective when only 10 µg/ml of each peptide was added (not shown). Plots show % of CD4+ or CD8+ T-cells producing IFNγ at culture end when restimulated with freshly thawed autologous PBMC either unpulsed (UP) or pulsed with the individual peptides. The ability to employ this culture method to successfully drive MUC1- and/or HER2-specific T-cells, both CD4+ and CD8+, was observed for four out of four patients at various stages of breast cancer. **B.** PBMC from a healthy donor cultured as in **Fig 1B**, exposed to a cocktail of SEA1+SEA2 peptides during culture in either 24 well cluster plates or scaled up in G-Rex 100M (1-liter) culture vessels. Panels show frequencies of SEA-specific CD4+ (**B1**) and CD8+ (**B2**) T-cells when assayed on day 19 of culture, as well as the fold increase in absolute numbers (gross expansion) (**B3**). This is representative of three biological replicates.
